# CRISPR/Cas9-Mediated Generation of Mutant Lines in *Medicago truncatula* Indicates a Symbiotic Role of *MtLYK10* during Nodule Formation

**DOI:** 10.3390/biology13010053

**Published:** 2024-01-19

**Authors:** Chun-Xiao Zhang, Ru-Jie Li, Laura Baude, Didier Reinhardt, Zhi-Ping Xie, Christian Staehelin

**Affiliations:** 1 State Key Laboratory of Biocontrol and Guangdong Key Laboratory of Plant Resources, School of Life Sciences, Sun Yat-sen University, Guangzhou 510275, China; 2 Department of Biology, University of Fribourg, 1700 Fribourg, Switzerland

**Keywords:** genome editing, LysM receptor kinase, plant tissue culture, symbiosis

## Abstract

**Simple Summary:**

Clustered regularly interspaced short palindromic repeats (CRISPR)/CRISPR-associated protein 9 (Cas9) technology has become an important research tool for targeted mutagenesis in plants. However, the construction of mutants in the model legume *Medicago truncatula* remains challenging as it requires the combination of an efficient CRISPR/Cas9-mediated gene editing system for explants and specific tissue culture techniques for regeneration to whole plants. Here, we describe the construction of *M. truncatula* mutants using the *Agrobacterium tumefaciens*-mediated transformation of leaf explants. Regeneration to whole plants was performed at high efficiency (80%), and a gene editing efficiency of up to 70% was reached in the obtained plants. CRISPR/Cas9-mediated gene editing was achieved for three *M. truncatula* genes. Three mutant lines with knockout mutations in the LysM receptor kinase gene *MtLYK10* were further characterized. Inoculation experiments with nitrogen-fixing *Sinorhizobium meliloti* bacteria indicated that root nodule formation was impaired in the constructed *MtLYK10* mutants. In conclusion, we show that the described CRISPR/Cas9 system is suitable for efficient gene editing in *M. truncatula* and that *MtLYK10* plays a role in nodule symbiosis.

**Abstract:**

CRISPR/Cas9 systems are commonly used for plant genome editing; however, the generation of homozygous mutant lines in *Medicago truncatula* remains challenging. Here, we present a CRISPR/Cas9-based protocol that allows the efficient generation of *M. truncatula* mutants. Gene editing was performed for the LysM receptor kinase gene *MtLYK10* and two major facilitator superfamily transporter genes. The functionality of CRISPR/Cas9 vectors was tested in *Nicotiana benthamiana* leaves by editing a co-transformed *GUSPlus* gene. Transformed *M. truncatula* leaf explants were regenerated to whole plants at high efficiency (80%). An editing efficiency (frequency of mutations at a given target site) of up to 70% was reached in the regenerated plants. Plants with *MtLYK10* knockout mutations were propagated, and three independent homozygous mutant lines were further characterized. No off-target mutations were identified in these *lyk10* mutants. Finally, the *lyk10* mutants and wild-type plants were compared with respect to the formation of root nodules induced by nitrogen-fixing *Sinorhizobium meliloti* bacteria. Nodule formation was considerably delayed in the three *lyk10* mutant lines. Surprisingly, the size of the rare nodules in mutant plants was higher than in wild-type plants. In conclusion, the symbiotic characterization of *lyk10* mutants generated with the developed CRISPR/Cas9 protocol indicated a role of *MtLYK10* in nodule formation.

## 1. Introduction

*Medicago truncatula* (barrel medic) is a model legume and particularly suitable for research on plant–microbe interactions such as the nodule symbiosis with nitrogen-fixing bacteria (rhizobia) and root colonization by symbiotic arbuscular mycorrhizal (AM) fungi. In the nodule symbiosis, *Sinorhizobium* (*Ensifer*) bacteria enter roots via root hairs, and nitrogen-fixing bacteroids in formed root nodules supply *M. truncatula* with fixed nitrogen [1]. In the AM symbiosis, *M. truncatula* and many other plants benefit from enhanced access to mineral nutrients supplied by the AM fungi, especially phosphorus [2]. *M. truncatula* is an annual and self-pollinating legume related to the pasture legume *M. sativa* (alfalfa) [3,4]. The genome of *M. truncatula* is diploid and has been sequenced for ecotypes such as Jemalong A17 [5] and R108 [6,7,8,9]. Various research tools such as mutant collections containing *Tnt1* retrotransposon insertions [10,11] and gene expression data for different tissues and mutants (e.g., MtExpress gene expression atlas [12]) are available. *M. truncatula* is amenable to genetic transformation using *Agrobacterium tumefaciens* carrying a given binary vector. The construction of stable transformants usually includes the regeneration of transformed leaf explants to whole plants [13,14,15,16], whereas floral dip transformation provides suboptimal results in *M. truncatula* [17]. Leaf explants of ecotype R108 exhibit high regeneration capacity and are thus most suitable for obtaining whole plants [13,14]. Furthermore, transgenic *M. truncatula* roots (hairy roots) can be obtained through transformation with the help of *Agrobacterium rhizogenes* strains [18,19].

Many bacteria and archaea possess an adaptive defense system that can act against viruses and other foreign DNA. Clustered regularly interspaced short palindromic repeats (CRISPR) derived from foreign DNA are incorporated into the prokaryotic genome and then transcribed. Then, the produced RNAs undergo processing steps resulting in short CRISPR RNAs (crRNAs) that guide CRISPR-associated (Cas) protein(s) to homologous nucleic acid target sequences. As a result, the prokaryotes can efficiently degrade undesired viral and other foreign DNA [20]. Taken advantage of these CRISPR-Cas systems, eukaryotic genomes can now be genetically modified, e.g., by the Cas9 nuclease gene of *Streptococcus pyogenes* in combination with a designed single guide RNA (sgRNA) construct. The sgRNA molecule consists of the target-specific crRNA fused to the trans-activating CRISPR RNA (tracrRNA). The Cas9-sgRNA complex is then guided to the desired target DNA region in the genome which contains a protospacer adjacent motif (PAM) required for cleavage by Cas9 [21]. The generated double-stranded DNA breaks are then repaired by cellular DNA repair mechanisms, often resulting in short deletions and/or insertions at the cleavage site [22]. CRISPR/Cas9-mediated gene editing has become a tool for targeted mutagenesis in plants [23], including *M. truncatula* transformed with the help of *A. tumefaciens* [24,25,26,27,28,29,30,31,32] or *A. rhizogenes* [33,34,35,36,37,38]. However, the generation of homozygous *M. truncatula* mutant lines remains challenging as it requires the combination of an efficient CRISPR/Cas9-mediated gene editing system for explants and specific tissue culture techniques for regeneration to whole plants.

Here, we describe the construction of *M. truncatula* mutants using the transformation of leaf explants with *A. tumefaciens* carrying constructed CRISPR/Cas9 binary vectors. Regeneration to whole plants was performed at high efficiency. Obtained *M. truncatula* lines with knockout mutations in the LysM receptor kinase gene *MtLYK10* [39] were further characterized. Inoculation experiments with nitrogen-fixing *S. meliloti* bacteria indicated that the described CRISPR/Cas9 system is suitable for efficient gene editing in *M. truncatula* and that *MtLYK10* plays a role in nodule symbiosis.

## 2. Materials and Methods

### 2.1. Bacterial Strains and Growth Media

*Escherichia coli* DH5α, Top10, and DH10B were grown in LB medium (5 g·L^−1^ yeast extract, 10 g·L^−1^ tryptone, 10 g·L^−1^ NaCl) at 37 °C. Strain DH5α was used for the construction of plasmids pUC-tracrRNA-P_MtU6-1_ and pUC-tracrRNA-P_MtU6-26_. Strain Top10 allowed the maintenance of plasmids containing the *ccdB* gene (pBS-*Cas9*-*ccdB*, pISV-*Cas9*P_35S_, and pISV-*Cas9*P_GmUbi_). Constructed CRISPR/Cas9 binary vectors were transformed into strain DH10B suitable for harboring large plasmids. *Agrobacterium tumefaciens* EHA105 [40] was grown in LB medium at 27 °C. *Sinorhizobium* (*Ensifer*) *meliloti* strain 1021 was cultured in TY medium (5 g·L^−1^ tryptone, 3 g·L^−1^ yeast extract, 0.4 g·L^−1^ CaCl_2_) at 27 °C. Details on plasmids used in this study are shown in Supplementary Materials (Table S1). Bacterial culture media were supplemented with appropriate antibiotics (100 mg·L^−1^ ampicillin, 50 mg·L^−1^ kanamycin, 25 mg·L^−1^ rifampicin, and 50 mg·L^−1^ streptomycin).

### 2.2. Plant Material, Seed Germination, and Plant Growth Conditions

*Medicago truncatula* Gaertn. ecotype R108 (also known as R108-1 and spp. *tricycla* R108), *Nicotiana benthamiana* Domin, and *Glycine max* (L.) Merr. cv. Willams 82 (soybean) were used in this study. For germination, *M. truncatula* seed coats were scarified with a needle. The seeds were surface-sterilized through immersion in diluted sodium hypochlorite (commercial bleach; ~1.0% (*w*/*v*) active chlorine) on a shaker for 10 min. After incubation, the seeds were washed several times with sterilized H_2_O. The seeds were then placed on 0.8% (*w*/*v*) water agar plates. The inverted plates were incubated at 4 °C in the dark for 48 h and then at 22 ± 1 °C for 18–20 h. The germinated seedlings were then planted into 2.5 L pots filled with soil containing vermiculite, expanded clay, and peat-based potting soil (Jiffy substrate TPS fine pH 5.8; Jiffy, Moerdijk, The Netherlands) at a ratio of 3:1:1 (*v*/*v*/*v*). The plants were grown in a growth room at 22 ± 1 °C under 16/8 h light/dark conditions (2000 lx light intensity; Philips Lifemax TL-D 36 W/54-765 and TL-D 36 W/29–530 daylight fluorescent tubes at a ratio of 3:1) [41]. *N. benthamiana* seeds were surface-sterilized with 70% (*v*/*v*) ethanol. The seedlings were planted into the Jiffy substrate and plants were kept in a temperature-controlled greenhouse at 25 ± 1 °C under 12/12 h light/dark conditions. *G. max* seeds were immersed in a 75% (*v*/*v*) ethanol solution for 2 min, washed several times with sterilized H_2_O, and then incubated in a 20% (*v*/*v*) H_2_O_2_ solution for 5 min. After washing with sterilized H_2_O, the seeds were incubated overnight at 4 °C and then placed onto 0.8% (*w*/*v*) water agar plates. The plates were incubated in the dark at 25 ± 1 °C for 3–4 days. The germinated seedlings were transferred into 8 L pots filled with vermiculite, expanded clay, and Jiffy substrate at a ratio of 3:1:2 (*v*/*v*/*v*). The plants were grown in a temperature-controlled greenhouse at 25 ± 1 °C under 16/8 h light/dark conditions.

### 2.3. Isolation of Genomic DNA from Plants

Plant DNA was extracted from leaves using the cetyltrimethylammonium bromide (CTAB) method [42]. Harvested leaf tissue (30–50 mg) was frozen with liquid nitrogen and ground in 1.5-mL microtubes using a plastic pestle. The powder was then mixed with 600 μL prewarmed (65 °C) CTAB extraction buffer (50 mM Tris-HCl pH 8.0, 0.6 M NaCl, 10 mM EDTA, 1% (*w*/*v*) CTAB, 0.1% (*v*/*v*) β-mercaptoethanol). The samples were then incubated at 65 °C for 30 min with gentle shaking. An equal volume of chloroform was then added and the microtubes were shaken well. Subsequently, a 2/3 volume of pre-chilled isopropanol was applied, and the samples were mixed gently. After incubation at −20 °C for 10 min, the microtubes were centrifuged (12,000 rpm; 10 min). The supernatant was discarded, and the precipitated DNA was washed twice with 70% (*v*/*v*) ethanol. Finally, the DNA was air-dried and then dissolved in H_2_O.

### 2.4. Construction of CRISPR/Cas9 Vectors

The constructed plasmids are shown in Supplementary Materials (Table S1) and the primers used are listed in Supplementary Materials (Table S2). Firstly, a DNA fragment containing *Cas9p* (plant codon optimized *Cas9* gene of *S. pyogenes* with an increased GC content) was PCR-amplified from pYLCRISPR/*Cas9*P_35S_-B [43]. The PCR mixtures (50 μL) contained 50 ng template DNA, 0.25 μM of primers 1 and 2, 0.2 mM dNTP, 1.5 mM MgSO_4_, 5 μL 10 × PCR Buffer, and 1 unit of KOD-Plus-Neo polymerase (Toyobo, Osaka, Japan). The PCR conditions were as follows: (i) 94 °C for 2 min; (ii) 35 cycles: 98 °C for 10 s, 68 °C for 2.5 min; and (iii) 68 °C for 10 min. The amplicon consisted of an enhancer sequencing, the coding region of *Cas9p* with N-terminal and C-terminal nuclear localization signal (NLS) sequences [44], and the cauliflower mosaic virus (CaMV) terminator. In parallel, primers 3 and 4 were used to amplify the *ccdB* expression cassette (with the *lac* promoter of *E. coli*) from pYLCRISPR/*Cas9*P_35S_-B. The CcdB toxin targets DNA gyrase and thus can be used for the positive selection of recombinant DNA [45]. The amplified *Cas9* and *ccdB* cassettes were then inserted into pBluescript II SK (+) (Stratagene, La Jolla, CA, USA), which was digested with *Sma*I and *Not*I using the One Step Cloning Kit from Novoprotein (Suzhou, China). The plasmid was named pBS-*Cas9*-*ccdB* and maintained in *E. coli* Top10.

To drive *Cas9p* expression under the control of a strong plant promoter, a soybean ubiquitin gene promoter sequence, known to be active in *M. truncatula* [24,27], was cloned from genomic *G. max* DNA (GenBank accession number EU310508.1; abbreviated as P_GmUbi_ in plasmids of this study). The PCR mixture (50 μL) contained 25 ng template DNA, 0.2 μM of primers 5 and 6, 200 mM dNTP, and 2.5 units of LA Taq polymerase (Takara Shuze, Osaka, Japan). The PCR conditions were as follows: (i) 95 °C for 5 min; (ii) 35 cycles: 95 °C for 30 s, 58 °C for 30 s, 72 °C for 1 min; and (iii) 72 °C for 10 min. The amplicon was inserted into the T-vector pMD19-T (Takara Shuze, Osaka, Japan) using the DNA ligase provided by the supplier. The resulting plasmid was named pMD19-P_GmUbi_.

*DsRed1* encoding a red fluorescent protein of the mushroom coral *Discosoma* sp. [46] was used to visualize the transformed *M. truncatula* plants. DNA was PCR-amplified from pRT104-*DsRed1* [47] with primers 7 and 8, and the reaction conditions were identical to those for the construction of pMD19-P_GmUbi_. The amplicon, consisting of a CaMV 35S promoter, *DsRed1*, and the polyA signal sequence of pRT104 [48], was cloned into pMD19-T. The *Bsa*I restriction site in *DsRed1* was then removed through site-directed mutagenesis [49] using KOD-Plus-Neo polymerase and the primers 9 and 10. *Dpn*I was then used to eliminate the template DNA. The obtained plasmid was named pMD19-*DsRed1*(ΔB). Subsequently, the *DsRed1*(ΔB) expression cassette was ligated into the *Hind*III site of the binary vector pISV2678, constructed by Michael Schultze at the CNRS (Gif-sur-Yvette, France). This vector is a derivative of pGPTV-BAR [50] and contains a *Bar* (bilaphos resistance) gene under the control of a nopaline synthase promoter for glufosinate selection in transformed plants. The pISV2678 vector carrying the *DsRed1*(ΔB) expression cassette was named pISV-*DsRed1*(ΔB). Except for the missing *Bsa*I site, pISV-*DsRed1*(ΔB) is identical to pISV-*DsRed1* constructed previously [47] (GenBank accession number MW701373; Addgene ID 171024).

The pISV-*DsRed1*(ΔB) vector was further modified to construct a vector containing *Cas9p* and *ccdB*. DNA from pBS-*Cas9*-*ccdB* was amplified with primers 11 (with a *Pac*I sequence) and 12, and the amplicon was cloned into the *EcoR*I site of pISV-*DsRed1*(ΔB). The resulting binary vector, named pISV-CRISPR/*Cas9*P_35S_, contained the double CaMV promoter from pISV-*DsRed1*(ΔB), a translational enhancer sequence (5′ leader of tobacco etch virus), the *Cas9p* gene sequence with a CaMV 35S terminator, and the *ccdB* expression cassette flanked by *Bsa*I restriction sites.

To obtain a similar vector with *Cas9p* driven by a ubiquitin gene promoter, the GmUbi promoter sequence amplified from pMD19-P_GmUbi_ (primers 5 and 6) was cloned into the generated *Pac*I site of pISV-CRISPR/*Cas9*P_35S_. The resulting plasmid was named pISV-CRISPR/*Cas9*P_GmUbi_.

Next, pUC18 [51] derivatives containing tracrRNA and small nuclear RNA (snRNA) promoter sequences of *M. truncatula* (P_MtU6-1_ and P_MtU6-26_) were constructed. DNA containing the pUC18 plasmid backbone and the tracrRNA was PCR-amplified from pYLsgRNA-AtU6-1 [43] using primers 13 and 14. The P_MtU6-1_ sequence was obtained from a previous publication [26]. The P_MtU6-26_ sequence of *M. truncatula* R108 was the result of a BLAST search at the *Medicago truncatula* Genome Database [52,53] using the P_AtU6-26_ sequence of *A. thaliana* [54] as a query sequence. The promoter sequences were amplified from the genomic DNA of *M. truncatula* R108 (primers 15 and 16 for P_MtU6-1_; primers 17 and 18 for P_MtU6-26_). The PCR mixtures (50 μL) contained 50 ng template DNA, 0.25 μM of each primer, 0.2 mM dNTP, 1.5 mM MgSO_4_, 5 μL 10 × PCR Buffer, and 1 unit of KOD-Plus-Neo polymerase. The PCR conditions were as follows: (i) 94 °C for 2 min; (ii) 98 °C for 10 s, 68 °C for 1.5 min; 35 cycles; and (iii) 68 °C for 10 min. The amplicons were then fused using a Gibson Assembly Cloning Kit (Novoprotein, Suzhou, China). The resulting plasmids containing two *Bsa*I restriction sites were named pUC-tracrRNA-P_MtU6-1_ and pUC-tracrRNA-P_MtU6-26_, respectively.

To design sgRNA sequences for a specific gene to be mutated, target sequences upstream of the PAM sequence (5′-NGG-3′) in a given gene sequence were identified using CRISPR-P 2.0 [55,56] and targetDesign [57,58]. In this study, target sequences were used for the *β*-glucuronidase (*GUSPlus*) gene of pCAMBIA1305 and three *M. truncatula* R108 genes [7]. In this article, these genes were named *MtLYK10* (*M. truncatula* lysin-motif receptor-like kinase 10, Medtr5g033490), *MtMFS1* (*M. truncatula* major facilitator superfamily transporter 1, Medtr3g093270), and *MtMFS2* (*M. truncatula* major facilitator superfamily transporter 2, Medtr3g093290). The synthesized target-specific 20-bp oligonucleotides (crRNA), are listed in Supplementary Materials (Table S3). The oligonucleotide pairs (1 μM in 20 μL water) were then annealed to form a double strand with ATTG/CAAA overhangs (samples heated to 90 °C for 1 min and then cooled to room temperature).

The sgRNA expression cassettes were constructed as follows. (i) In a restriction-ligation reaction, the double-stranded oligonucleotide (crRNA) with the ATTG/CAAA overhangs was fused with tracrRNA-P_MtU6-1_ or tracrRNA-P_MtU6-26_ DNA digested with *Bsa*I. The mixture (10 μL) contained 5 U *Bsa*I-HFV2 (NEB, Ipswich, MA, USA), 1 μL 10 × *Bsa*I reaction buffer, 20 U T4 ligase (Genestar, Beijing, China), 1 μL 10 × T4 ligation buffer, 20 ng plasmid DNA (pUC-tracrRNA-P_MtU6-1_ or pUC-tracrRNA-P_MtU6-26_), and 0.1 μM of a double-stranded oligonucleotide. The mixture was subject to 5 thermal cycles (37 °C for 5 min; 20 °C for 5 min). (ii) Then, the ligation product (3 μL) served as a template for a PCR to amplify a given sgRNA expression cassette containing the P_MtU6-1_ or P_MtU6-26_ promoter, the tracrRNA sequence, and crRNA sequence. The reaction mixture (25 μL) contained 0.2 μM of primers 19 and 20, 0.5 U of KOD-Plus-Neo polymerase, 2.5 μL 10 × PCR Buffer, 0.2 mM dNTPs, and 1.5 mM MgSO_4_. The PCR was performed for 20 cycles (98 °C for 15 s, 58 °C for 15 s, 68 °C for 10 s). (iii) A total of 1 μL of the PCR product was then directly used as template for a second PCR (50 μL) to produce sgRNA expression cassettes flanked by *Bsa*I restriction sites (primers 21 and 22 for sgRNA expression cassettes containing the P_MtU6-1_ promoter; primers 23 and 24 for sgRNA expression cassettes containing the P_MtU6-26_ promoter). The reaction mixture contained 0.2 μM of each primer, 1 U of KOD-Plus-Neo polymerase; 5 μL 10 × PCR Buffer, 0.2 mM dNTPs, and 1.5 mM MgSO_4_ (98 °C for 15 s, 58 °C for 15 s, 68 °C for 10 s; 25 cycles). (iv) Using Golden Gate cloning [59], the constructed sgRNA expression cassettes with terminal *Bsa*I restriction sites were assembled and inserted into a constructed binary vector containing a *Cas9p* expression cassette (pISV-CRISPR/*Cas9*P_35S_ and pISV-CRISPR/*Cas9*P_GmUbi_). For comparison, pYLCRISPR/*Cas9*P_35S_-B containing the pCAMBIA1300 backbone, *Cas9p*, under the control of a double CaMV promoter [43] was also used in initial studies. Each reaction mixture (10 μL), containing 60 ng DNA of the binary vector, 15 ng DNA of a given sgRNA expression cassette, 10 U *Bsa*I-HFV2, and 1 μL 10 × *Bsa*I reaction buffer, was incubated at 37 °C for 30 min. Subsequently, 35 U of T4 DNA ligase and 1 μL 10 × T4 ligation buffer were added and the samples were incubated at 37 °C for 5 min, 10 °C for 5 min and 20 °C for 5 min for a total of 15 cycles. Using the described procedure, two sgRNA expression cassettes, each with different promoters (P_MtU6-1_ and P_MtU6-26_, respectively), were assembled and ligated into the binary vector. Finally, the ligation products were transformed into competent *E. coli* DH10B cells which do not grow when transformed, with plasmids containing the *ccdB* expression cassette. The insertion of the sgRNA expression cassettes into the binary vectors was confirmed through sequencing (pYLCRISPR/*Cas9*P_35S_-sgRNA(*GUS*), pISV-CRISPR/*Cas9*P_35S_-sgRNA(*GUS*), pISV-CRISPR/*Cas9*P_GmUbi_-sgRNA(*GUS*), pISV-CRISPR/*Cas9*P_GmUbi_-sgRNA(*LYK10*), pISV-CRISPR/*Cas9*P_35S_-sgRNA(*MFS1*/*MFS2*), and pISV-CRISPR/*Cas9*P_GmUbi_-sgRNA(*MFS1*/*MFS2*)).

### 2.5. Electroporation of CRISPR/Cas9 Binary Vectors into A. tumefaciens

The constructed CRISPR/Cas9 binary vectors were transformed into competent *A. tumefaciens* EHA105 cells (Weidi, Shanghai, China) with the help of an electroporator (Ding Guo, Guangzhou, China). Pre-cooled cells (100 μL) were carefully supplemented with vector DNA (≈0.5 μg) in a sterilized 0.2 cm electroporation cuvette. After electroporation at 1500 V for 10 ms, the cells were manually mixed with 1 mL of LB medium (27 °C) and transferred to 1.5 mL plastic tubes. The bacteria were placed on a shaker (200 rpm, 27 °C) for 3 h, and 100 μL of each bacterial suspension was then spread on LB agar plates containing appropriate antibiotics (50 mg·L^−1^ kanamycin and 25 mg·L^−1^ rifampicin). After incubation (27 °C) for two days, single colonies were picked from the plates. The presence of the vectors in the bacteria was confirmed via PCR using primers listed in Supplementary Materials (Table S2).

### 2.6. Agrobacterium-Mediated Transformation of N. benthamiana Leaves

To test the functionality of the different CRISPR/Cas9 vectors binary vectors, they were examined for their capacity to induce mutations in the *GUSPlus* gene expressed in *N. benthamiana* leaf cells transformed with agrobacteria. The binary vector pCAMBIA1305 containing *GUSPlus* with a CaMV 35S promoter was used in these experiments. GUS activity in transformed tissue was visualized using histochemical staining with 5-bromo-4-chloro-3-indolyl β-D-glucuronide (X-Gluc) [60]. For *Agrobacterium*-mediated transformation, individual colonies of *A. tumefaciens* EHA105 containing pCAMBIA1305, pYLCRISPR/*Cas9*P_35S_-sgRNA(*GUS*), pISV-CRISPR/*Cas9*P_35S_-sgRNA(*GUS*), and pISV-CRISPR/*Cas9*P_GmUbi_-sgRNA(*GUS*) were grown in 5 mL of LB medium (supplemented with 50 mg L^−1^ kanamycin and 25 mg L^−1^ rifampicin) overnight at 27 °C. The cultures were then harvested using centrifugation (6000 rpm for 5 min), washed with 10 mM MgCl_2_, and then resuspended in the infiltration solution (10 mM MgCl_2_ supplemented with 200 μM acetosyringone) to obtain an OD_600_ ≈ 0.1. Next, the resuspended culture was shaken at 50 rpm (27 °C, in the dark) for 3 h. Each strain containing a CRISPR/Cas9 binary vector was then mixed with an equal volume of the strain carrying pCAMBIA1305. The bacterial suspensions were then infiltrated into the underside of 4–5-week-old *N. benthamiana* leaves using a 1-mL syringe (without needle). The plants were kept in a temperature-regulated greenhouse and leaves were harvested 7 days post infiltration [61].

### 2.7. Transformation of M. truncatula Leaf Explants and Regeneration to Whole Plants

*M. truncatula* R108 leaf explants were transformed with *A. tumefaciens* EHA105 carrying pISV-CRISPR/*Cas9*P_GmUbi_-sgRNA(*LYK10*), pISV-CRISPR/*Cas9*P_35S_-sgRNA(*MFS1*/*MFS2*), or pISV-CRISPR/*Cas9*P_GmUbi_-sgRNA(*MFS1*/*MFS2*), according to previously published procedures with various modifications [13,14,15,16]. A single bacterial colony carrying a given CRISPR/Cas9 binary vector was inoculated into 4 mL of LB medium supplemented with 50 mg·L^−1^ kanamycin and 25 mg·L^−1^ rifampicin. The bacteria were cultured on a shaker (200 rpm, 27 °C) for 24 h, and 1 mL of the preculture was transferred into 30 mL LB medium containing the same antibiotics. The cells were then cultured on the same shaker for 12–16 h until the cells reached an OD_600_ ≈ 1. The cells were centrifuged at 4000 rpm for 15 min and then resuspended (OD_600_ = 0.3) in liquid SH-1 medium, which is defined as SH medium (0.185 g·L^−1^ MgSO_4_·7H_2_O, 2.83 g·L^−1^ KNO_3_, 0.463 g·L^−1^ (NH_4_)_2_SO_4_, 0.4 g·L^−1^ KH_2_PO_4_, 0.166 g·L^−1^ CaCl_2_·2H_2_O, 1 mg·L^−1^ MnSO_4_·H_2_O, 0.5 mg·L^−1^ H_3_BO_3_, 0.1 mg·L^−1^ ZnSO_4_·7H_2_O, 0.1 mg·L^−1^ KI, 0.1 mg·L^−1^ Na_2_MO_4_·2H_2_O, 0.2 mg·L^−1^ CuSO_4_·5H_2_O, 0.1 mg·L^−1^ CoCl_2_·6H_2_O, 0.2 mg·L^−1^ CuSO_4_·5H_2_O, 5 mg·L^−1^ nicotinic acid, 5 mg·L^−1^ thiamine HCl, 5 mg·L^−1^ pyridoxine HCl, 140 mg·L^−1^ FeNa-EDTA, and 100 mg·L^−1^ myo-inositol; [62]) supplemented with 30 g·L^−1^ sucrose, 4 mg·L^−1^ 2,4-dichlorophenoxyacetic acid (2,4-D), and 0.5 mg·L^−1^ 6-benzylaminopurine (BAP). The SH-1 medium was adjusted to pH 5.8 with KOH. Healthy leaves of 4–6-week-old *M. truncatula* R108 plants were surface-sterilized with diluted sodium hypochlorite solution (≈0.6% (*w*/*v*) active chlorine) for 15 min and then washed several times with sterilized H_2_O. The leaves were cut into square pieces (≈1 cm^2^) with a sharp scalpel blade and immersed in the prepared *A. tumefaciens* suspension. For agroinfiltration, the bacterial suspension with the immersed leaf explants was exposed to a vacuum (≈−0.085 MPa) for 10 min. The explants were then gently shaken (60 rpm, room temperature) for 1 h, dried with autoclaved filter papers, and transferred to solid SH-1 medium plate containing 3 g·L^−1^ phytagel. After 3 days of co-cultivation, the explants were washed in liquid SH-1 medium for 20 min, dried with a sterilized filter paper, and finally transferred to solid SH-2 medium, which is defined as SH-1 medium supplemented with 300 mg·L^−1^ timetin (to suppress the growth of agrobacteria) and 3 mg·L^−1^ of the herbicide glufosinate (phosphinothricin) ammonium salt. Glufosinate allows selection for transgenic *M. truncatula* cells expressing the *Bar* gene, which confers resistance to glufosinate. The plates were kept at 24 °C in the dark. The explants were transferred every two weeks to identical plates that were freshly prepared, and the formation of calli was observed at 6–7 weeks post agroinfiltration. About 4–5 weeks later, the calli were transferred to plates containing solid MSBK plates (pH 5.8), which is defined as 4.43 g·L^−1^ Murashige and Skoog (MS) basal medium (Coolaber Beijing, China; [63]) supplemented with 30 g·L^−1^ sucrose, 1 mg·L^−1^ kinetin, 0.5 mg·L^−1^ BAP, 2 mg·L^−1^ glufosinate, 300 mg·L^−1^ timetin, and 3 g·L^−1^ phytagel [16]. The plates were incubated at 22 ± 1 °C under 16/8 h light/dark conditions (2000 lx light intensity; Philips Lifemax TL-D 36 W/54-765 and TL-D 36 W/29–530 daylight fluorescent tubes at a ratio of 3:1). About two weeks later, green somatic pro-embryos were observed and transferred to plates filled with phytohormone-free SH-3 medium (SH medium supplemented with 20 g·L^−1^ sucrose, 150 mg·L^−1^ timetin, 2 mg·L^−1^ glufosinate, and 3 g·L^−1^ phytagel). The plates were refreshed every two weeks. Plantlets with fully expanded trifoliate leaves were observed after 7–8 weeks growth on these plates. The plantlets were then transferred to 350-mL glass vessels which were partially filled with ≈150 mL SH-3 medium. To ensure gas exchange, the lids of the glass vessels had a hole of 0.79 cm^2^, which was covered with filter paper. The plantlets were placed into fresh glass vessels every two weeks. Fully regenerated plants with well-developed roots were obtained after about 10 weeks growth in these vessels. Finally, the plants were transferred to sterilized 300-mL plastic jar units linked with a cotton wick (0.5 cm in diameter). The upper jar contained vermiculite and expanded clay (3:1 *v*/*v*), and the lower jar was filled with half-strength B&D [64] nutrient solution (125 μM CaCl_2_·2H_2_O, 62.5 μM KH_2_PO_4_, 1.25 μM Fe-citrate, 31.25 μM MgSO_4_·7H_2_O, 187.5 μM K_2_SO_4_, 0.125 μM MnSO_4_·7H_2_O, 0.25 μM H_3_BO_3_, 0.025 μM CuSO_4_·5H_2_O, 0.0125 μM CoSO_4_·7H_2_O, 0.0125 μM Na_2_MoO_4_·2H_2_O, and 0.0625 μM ZnSO_4_·H_2_O) supplemented with 1 mM KNO_3_. The upper jar was covered with a light transparent lid for several days to acclimatize the plants to the new environment. The jar units were kept in a temperature-controlled growth room as described above.

Plants of the T_0_ generation were examined for the emission of red fluorescent signals. For seed production, the transgenic plants were transferred to 2.5 L pots filled with vermiculite and expanded clay (3:1 *v*/*v*). The plants were watered with half-strength B&D nutrient solution containing 0.5 mM KNO_3_ (≈300 mL per pot every 3 days).

### 2.8. Detection of Red Fluorescent Signals in Transformed M. truncatula Plants

A Zeiss ImagerZ1 fluoresce microscope (Carl Zeiss, Oberkochen, Germany) was used to detect red fluorescent signals in transgenic *M. truncatula* plants expressing *DsRed1* [46]. The excitation wavelengths were 540–552 nm and the emission window 575–640 nm. Typically, small root pieces were harvested from T_0_ generation plants before they were transferred from jar units to pots.

### 2.9. Identification of Mutations in Transformed Plants

Genomic DNA was extracted from the infiltration zone of *N. benthamina* leaves (5 leaves from 5 different plants per *A. tumefaciens* strain) to identify sgRNA-mediated mutations in *GUSPlus* sequences. Likewise, leaves of 10 transformed and regenerated *M. truncatula* plants were used for the extraction of genomic DNA to detect mutations in the *MtLYK10*, *MtMFS1*, and *MtMFS2* genes. The DNA was then used as a template for a PCR (primers 31 and 32 for *GUSPlus*; primers 33 and 34 for *MtMFS1*; primers 35 and 36 for *MtMFS2*; and primers 37 and 38 for *MtLYK10*). The reaction mixture (50 μL) contained 200 ng template DNA, 0.25 μM of gene-specific primers, 0.8 mM dNTP, 5 mM MgSO_4_, 5 μL 10 × PCR Buffer, and 2.5 U of LA Taq (2 × LA taq mix; Takara, Osaka, Japan). The annealing temperature was 60 °C and the extension time was 1 min. PCR products amplified from *M. truncatula* DNA were then directly subjected to Sanger sequencing. When the sequencing results showed double peak signals, the corresponding amplicon was inserted into pMD19-T and sequencing data with single peak signals were obtained from transformed *E. coli* colonies. PCR products amplified from *N. benthamiana* leaves were directly cloned into pMD19-T. For each transformed leaf, three randomly sampled *E. coli* colonies were sequenced to estimate the mutation frequency at the *GUSPlus* target sites.

### 2.10. Analysis of Generated lyk10 Mutant Plants

T_0_ generation plants with identified mutations in *MtLYK10* were propagated by selfing. The resulting T_1_ generation plants were then subjected to PCRs (primers 37 and 38; Supplementary Materials, Table S2) and sequencing to identify homozygous (biallelic) mutant plants in three independent *lyk10* mutant lines (*lyk10-1*, *lyk10-1*, and *lyk10-1*). The prediction of possible off-target mutations in the *lyk10* mutants was performed with offTarget software [57,65] using the corresponding crRNA-PAM sequences as query sequences. The nucleotide sequences of possible off-target regions in various genes (MTR_2g063570, MTR_7g029350, MTR_4g127480, MTR_2g069950, MTR_5g024910, MTR_3g112020, MTR_1g054795, MTR_4g116370, and MTR_7g056073) were analyzed by sequencing the amplicons of PCRs using primers 41–58.

Furthermore, the nodule formation of homozygous *lyk10* mutant plants was analyzed. T_1_ generation plants (25–30 per identified mutation) and wild-type plants were grown in sterilized 300 mL plastic jar units (two jars were connected with a cotton wick). The upper jar was filled with vermiculite and expanded clay at a ratio of 3:1 (*v*/*v*). The lower jar contained B&D nutrient solution [64] (250 μM CaCl_2_·2H_2_O, 125 μM KH_2_PO_4_, 2.5 μM Fe-citrate, 62.5 μM MgSO_4_·7H_2_O, 375 μM K_2_SO_4_, 0.25 μM MnSO_4_·7H_2_O, 0.5 μM H_3_BO_3_, 0.05 μM CuSO_4_·5H_2_O, 0.025 μM CoSO_4_·7H_2_O, 0.025 μM Na_2_MoO_4_·2H_2_O, and 0.125 μM ZnSO_4_·H_2_O) supplemented with 1 mM KNO_3_. The growth conditions were the same as described above. After one week, plants (1 plant per jar unit) were inoculated with *S. meliloti* 1021. The bacteria were centrifuged and resuspended in 10 mM MgSO_4_ (OD_600_ adjusted to 0.2). Each jar system was inoculated with 2 mL bacteria. Genomic DNA was isolated from a harvested leaf at 7 days post inoculation (dpi) to identify homozygous *lyk10* mutant plants using sequencing, as described above. The roots of three homozygous mutant lines were harvested at two different time points (10 and 21 dpi) to determine the number of formed nodules for each plant. Longitudinal sections of nodules (harvested at 21 dpi) were analyzed using a Zeiss Lumar.V12 microscope (Carl Zeiss, Oberkochen, Germany). Image J2 software [66] was used to calculate the area of the sectioned nodules. Statistical analysis (Student’s *t*-test) was performed with GraphPad Prism (version 8.0.2 (263); Prism software, Vestal, NY, USA).

## 3. Results

### 3.1. Construction of CRISPR/Cas9 Binary Vectors Suitable for M. truncatula Transformation

**Figure 1:**
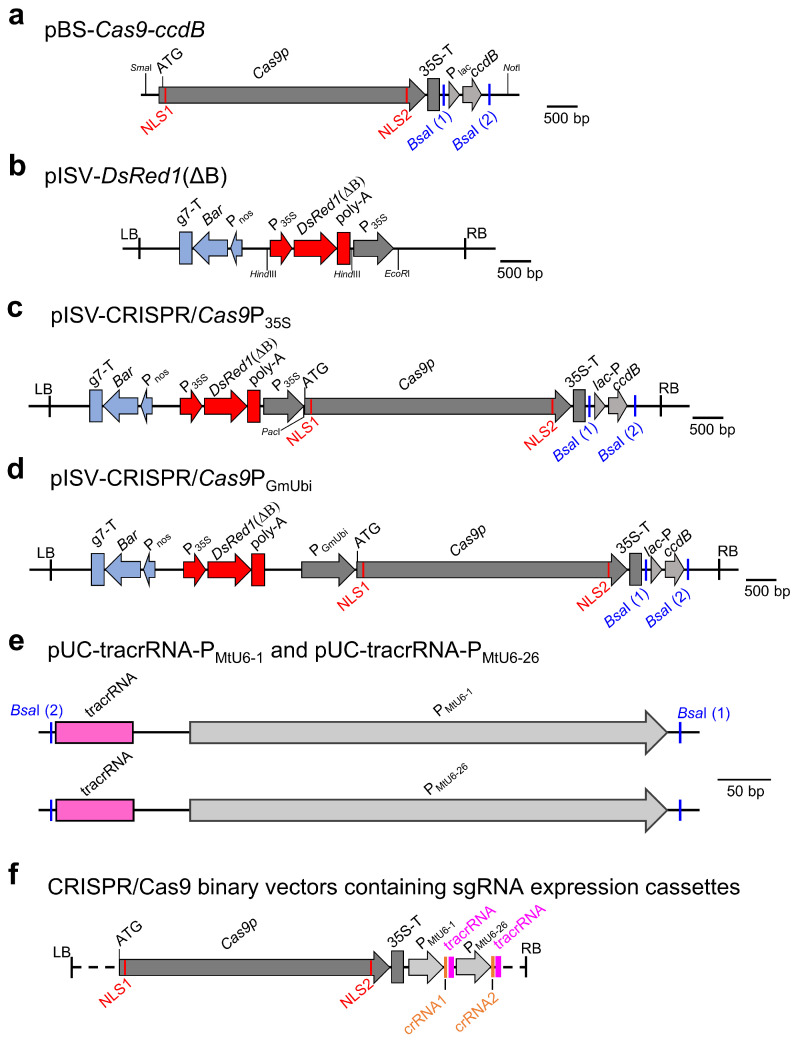
CRISPR/Cas9 plasmids constructed in this study. (**a**) Insert of pBS-*Cas9*-*ccdB* containing the *Cas9p* gene and the *ccdB* expression cassette flanked by *Bsa*I restriction sites. (**b**) T-DNA region of pISV-*DsRed1*(ΔB) containing the *Bar* gene expression cassette, the *DsRed1*(ΔB) expression cassette, and a double CaMV promoter. (**c**) T-DNA region of pISV-CRISPR/*Cas9*P_35S_ which is a pISV-*DsRed1*(ΔB) derivative containing *Cas9p* driven by a double CaMV promoter and the *ccdB* expression cassette flanked by *Bsa*I restriction sites. (**d**) T-DNA region of pISV-CRISPR/*Cas9*P_GmUbi_ which is a pISV-*DsRed1*(ΔB) derivative containing *Cas9p* driven by a GmUbi promoter and the *ccdB* expression cassette flanked by *Bsa*I restriction sites. (**e**) Inserts of pUC plasmids containing the tracrRNA sequence and a snRNA gene promoter of *M. truncatula* (P_MtU6-1_ or P_MtU6-26_) flanked by *Bsa*I restriction sites. (**f**) Schematic view of CRISPR/Cas9 binary vectors containing two sgRNA expression cassettes, each consisting of a tracrRNA and a crRNA sequence. Abbreviations: NLS, nuclear localization sequence; P: promoter; T: terminator; *ccdB*: suicide gene; *Bar*, phosphotransferase (PPT) gene; tracrRNA: trans-activating crRNA; crRNA: CRISPR RNA.

In previous studies, pISV2678, a binary vector with a *Bar* gene, was found to be very effective for the *A. tumefaciens*-mediated transformation of *M. truncatula* leaf explants [41,67,68,69]. We therefore aimed to construct a CRISPR/Cas9 vector with a pISV2678 backbone and a *DsRed1* expression cassette for the visual confirmation of transformants. The *Cas9p* gene with N-terminal and C-terminal NLS sequences was PCR-amplified from pYLCRISPR/*Cas9*P_35S_-B [43] along with the transcriptional enhancer 5’UTR of Tobacco Etch Virus and the CaMV terminator. Furthermore, a *ccdB* expression cassette was amplified from pYLCRISPR/*Cas9*P_35S_-B. Both amplicons were then cloned into pBluescript II SK (+), resulting in pBS-*Cas9*-*ccdB* (Figure 1a). Next, the binary vector pISV2678 was modified by inserting a *DsRed1* expression cassette (lacking the undesired *Bsa*I restriction site) into the *Hind*III site of pISV2678, resulting in pISV-*DsRed1*(ΔB) (Figure 1b). The insert of pBS-*Cas9*-*ccdB* was then cloned into the *EcoR*I site of pISV-*DsRed1*(ΔB), yielding pISV-CRISPR/*Cas9*P_35S_ (Figure 1c). In this vector, the *Cas9p* gene expression is under the control of a double CaMV promoter, while the *ccdB* expression cassette is flanked by unique *Bsa*I restriction sites. In addition, a similar binary vector, pISV-CRISPR/*Cas9*P_GmUbi_, was constructed in which *Cas9p* gene expression is driven by a strong ubiquitin gene promoter of soybean (Figure 1d). Furthermore, plasmids containing tracrRNA and snRNA promoter sequences of *M. truncatula* (P_MtU6-1_ or P_MtU6-26_) were constructed. The pUC18 plasmid backbone and the tracrRNA sequence were PCR-amplified from pYLsgRNA-AtU6-1 [43] and the P_MtU6-1_ or P_MtU6-26_ promoter sequences from genomic DNA of *M. truncatula*. The plasmids containing the amplified DNA fragments were named pUC-tracrRNA-P_MtU6-1_ and pUC-tracrRNA-P_MtU6-26_, respectively (Figure 1e).

Using CRISPR-P 2.0 and targetDesign software, target sequences upstream of the PAM sequence (5′-NGG-3′) were identified for the *GUSPlus* gene in the binary vector pCAMBIA1305 and the *M. truncatula* genes *MtLYK10*, *MtMFS1*, and *MtMFS2*. Synthesized oligonucleotides (listed in Supplementary Material, Table S3) were then annealed to form double strands with ATTG/CAAA overhangs (crRNA), which were then cloned into pUC-tracrRNA-P_MtU6-1_ or pUC-tracrRNA-P_MtU6-26_ digested with *Bsa*I. The obtained DNA was used as a template for a PCR to amplify a given sgRNA expression cassette consisting of the P_MtU6-1_ or P_MtU6-26_ promoter, the tracrRNA, and a synthesized crRNA. In a second PCR, terminal sequences with a *Bsa*I restriction site were introduced into the sgRNA expression cassette. Finally, using a restriction-ligation reaction with *Bsa*I, the sgRNA expression cassettes were inserted into pISV-CRISPR/*Cas9*P_35S_ (sgRNA expression cassettes to edit *GUSPlus*, *MtMFS1*, and *MtMFS2*) and pISV-*Cas9*P_GmUbi_ (sgRNA cassettes to edit *GUSPlus*, *MtLYK10*, *MtMFS1*, and *MtMFS2*), respectively. For comparison, pYLCRISPR/*Cas9*P_35S_-B [43], a pCAMBIA1300 derivative containing *Cas9p* under the control of a double CaMV 35S promoter, was also used in initial *GUSPlus* experiments. The procedure for cloning sgRNA expression cassettes and inserting them into the binary vectors is illustrated in Supplementary Materials (Figure S1). A schematic view of the obtained CRISPR/Cas9 binary vectors is shown in Figure 1f. All constructed binary vectors contained two sgRNA expression cassettes with different promoters (P_MtU6-1_ and P_MtU6-26_, respectively).

### 3.2. Testing for the Functionality of Constructed CRISPR/Cas9 Binary Vectors in N. benthamiana

**Figure 2:**
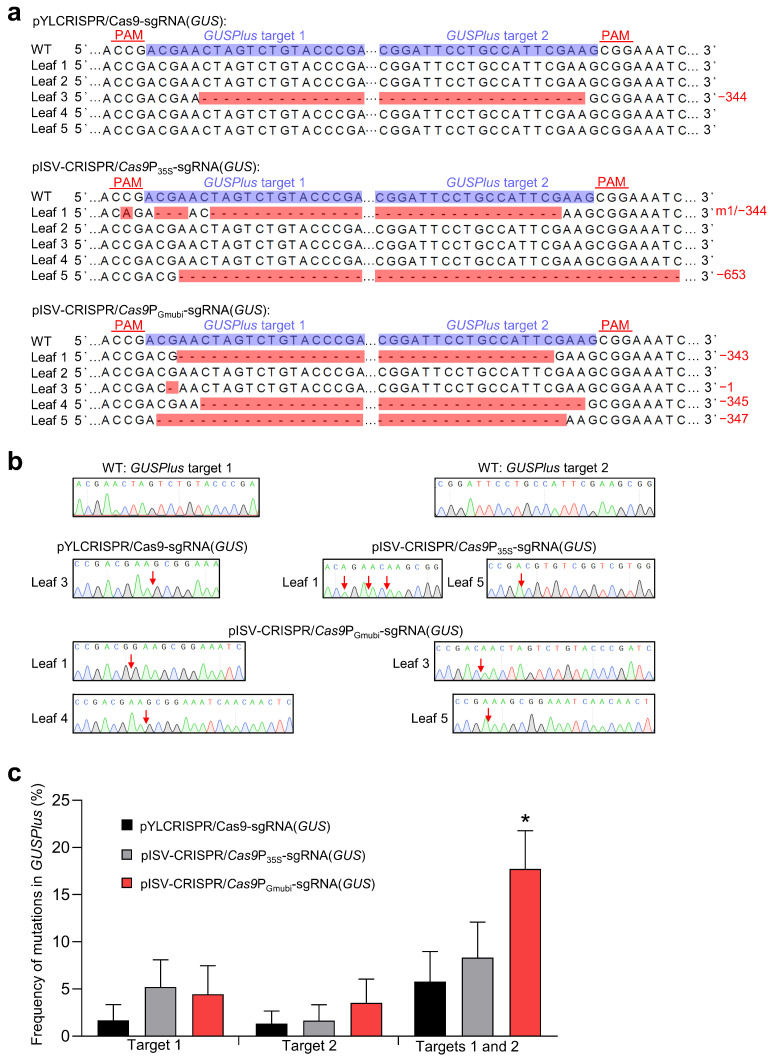
Testing *A. tumefaciens* strains carrying different CRISPR/Cas9 vectors in *N. benthamiana* leaves. The vectors pYLCRISPR/*Cas9*P_35S_-sgRNA(*GUS*), pISV-CRISPR/*Cas9*P_35S_-sgRNA(*GUS*), and pISV-CRISPR/*Cas9*P_GmUbi_-sgRNA(*GUS*) were examined for their capacity to induce mutations at two target sites in the co-transformed *GUSPlus* gene. (**a**) Sequencing results of DNA from five transformed leaves. The target sites besides the PAM sequence are shown in the wild-type (WT) sequence. Mutations at both target sites resulted in large deletions. Abbreviations: −, deletion; m, mutation. (**b**) Sanger sequencing chromatograms obtained from clones transformed into *E. coli*. The arrows mark the mutation sites. (**c**) Frequency of mutations at target sites 1 and 2 in *GUSPlus*. In total, 194 *GUSPlus* sequences were analyzed. Data indicate means ± SE (*n* = 5). The asterisk indicates a significantly increased mutation frequency induced by pISV-CRISPR/*Cas9*P_GmUbi_-sgRNA(*GUS*) as compared to the other binary vectors (Student’s *t*-test, *p* < 0.05).

Transient gene expression experiments with *N. benthamiana* plants were performed to examine the functionality of the constructed CRISPR/Cas9 binary vectors. The vectors (pISV-CRISPR/*Cas9*P_35S_-sgRNA(*GUS*) and pISV-CRISPR/*Cas9*P_GmUbi_-sgRNA(*GUS*)) contained two sgRNA expression cassettes to induce mutations at two different target sites of a co-transformed *GUSPlus* gene. For comparison, the two sgRNA expression cassettes targeting *GUSPlus* were also introduced into pYLCRISPR/*Cas9*P_35S_-B [43] (vector pYLCRISPR/*Cas9*P_35S_-sgRNA(*GUS*)). Prior to agroinfiltration, suspensions of *A. tumefaciens* EHA105 harboring a given CRISPR/Cas9 binary vector were mixed with an equal volume of EHA105 carrying pCAMBIA1305, which contains a *GUSPlus* expression cassette. Each bacterial suspension was injected into five leaves of five different *N. benthamiana* plants and leaves were harvested 7 days post infiltration. GUS activity tests with X-Gluc showed blue coloration in the infiltration zones, indicating the successful transformation of the *GUSPlus* gene. Subsequently, DNA was isolated from the infiltration zones and used as a template to amplify a *GUSPlus* gene fragment containing the two target sites. As the sequencing of amplicons resulted in double peaks, the amplicons were cloned into pMD19-T, which was then transformed into *E. coli* cells to obtain single peaks from isolated plasmid DNA. Sequence comparisons showed that all the CRISPR/Cas9 binary vectors tested can cause mutations at the *GUSPlus* target sites. In a number of sequences, gene knockout was observed due to a frameshift mutation at a single target site. Mutations at two target sites led to DNA deletion between the target sites. Examples of obtained *GUSPlus* sequence variants are shown in Figure 2a and the corresponding Sanger sequencing chromatograms in Figure 2b. The editing efficiency was then quantified for each binary vector using data from a total of 194 sequences. The use of the pISV-CRISPR/*Cas9*P_GmUbi_-sgRNA(*GUS*) vector resulted in the highest frequency of simultaneous mutations at both target sites (Figure 2c). These data indicated that the constructed CRISPR/Cas9 binary vectors were functional and thus could be used for experiments to edit specific genes in the *M. truncatula* genome.

### 3.3. Efficient CRISPR/Cas9-Mediated Generation of Mutant Lines in M. truncatula

**Figure 3:**
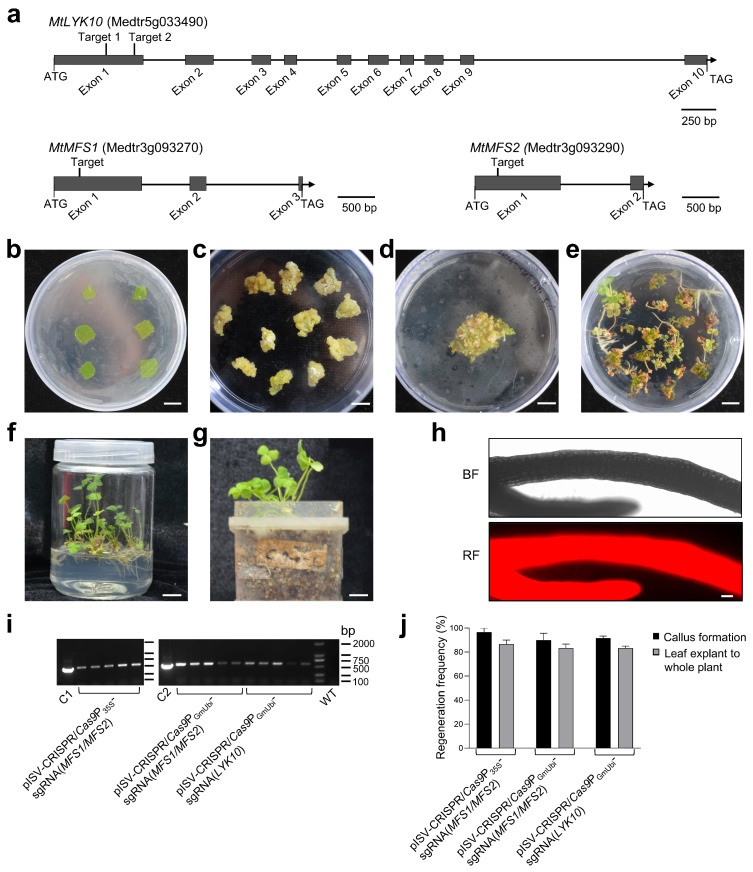
Transformation of leaf explants and regeneration to whole *M. truncatula* plants. (**a**) Schematic diagram illustrating the exon/intron structure of the *M. truncatula* genes *MtLYK10*, *MtMFS1*, and *MtMFS2*. The target sites designed for gene editing are in exon 1. (**b**) Explants from *M. truncatula* leaves after transformation with *A. tumefaciens* bacteria on SH-1 medium. (**c**) Six-week-old calli kept on SH-2 medium. (**d**) Calli placed on MSBK medium for 2 weeks. (**e**) Plantlets grown on SH-3 medium for 7 weeks. (**f**) Plants grown in glass vessels containing SH-3 medium for 10 weeks. (**g**) Plants grown in plastic jar units filled with vermiculite and expanded clay. DNA from individual plants was used for PCRs and sequencing. (**h**) Analysis for red fluorescent signals in roots using a Zeiss ImagerZ1 fluorescence microscope. Root pieces were taken from regenerated plants before transfer into plastic jar units. (**i**) PCR analysis of the *Bar* gene in plants transformed with the indicated binary vectors. The leaf DNA of *M. truncatula* wild-type plants (WT) served as a negative control and the plasmid DNA of pISV-CRISPR/*Cas9*P_35S_ and pISV-CRISPR/*Cas9*P_GmUbi_ as a positive control (lanes C1 and C2, respectively). (**j**) Regeneration efficiency of leaf explants transformed with the indicated binary vectors to form a callus or a whole plant. Scale bars: 1 cm in (**b**–**g**) and 100 μm in (**h**).

To determine whether specific *M. truncatula* genes can be mutated using the established CRISPR/Cas9 system, we constructed binary vectors to edit three *M. truncatula* genes, namely, the LysM-type receptor kinase gene *MtLYK10* [39] and two MFS transporter genes related to *N*-acetylglucosamine transporters in rice [70]. The exon/intron structure and selected target sites in these three *M. truncatula* genes are illustrated in Figure 3a. The constructed CRISPR/Cas9 binary vectors (pISV-CRISPR/*Cas9*P_35S_-sgRNA(*MFS1*/*MFS2*), pISV-CRISPR/*Cas9*P_GmUbi_-sgRNA(*MFS1*/*MFS2*), and pISV-CRISPR/*Cas9*P_GmUbi_-sgRNA(*LYK10*)) were then used for the *A. tumefaciens*-mediated transformation of leaf explants. Pictures illustrating the different cultivation steps from leaf disks to whole transgenic plants are shown in Figure 3b–g. The whole procedure lasted 6–9 months. The expression of transformed *DsRed1* in regenerated glufosinate-resistant plants (T_0_ generation) was confirmed through the fluorescence microscopy analysis of the roots (Figure 3h). A PCR analysis confirmed that the transformed plants contained the *Bar* gene (Figure 3i). A quantitative analysis indicated that most leaf disks could form a callus (callus formation efficiency of about 95%). The regeneration efficiency from calli to whole plants was also high. On average, transgenic plants could be obtained from about 85% of the calli (Figure 3j). The sequencing results of PCR-amplified DNA from the leaves of T_0_ generation plants indicated double peaks in the target regions, indicating that one allele of the target gene was successfully edited (Supplementary Materials, Figure S2). For confirmation, the amplicons were cloned into pMD19-T and sequenced. Only single peaks were observed in chromatograms obtained from the plasmid DNA of individual *E. coli* colonies.

**Figure 4:**
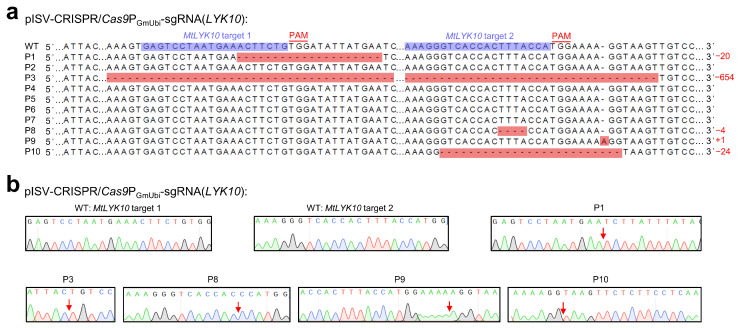
CRISPR/Cas9-mediated editing of *MtLYK10*. *M. truncatula* leaf explants were transformed with *A. tumefaciens* carrying pISV-CRISPR/*Cas9*P_GmUbi_-sgRNA(*LYK10*). Leaf DNA was extracted from wild-type plants and regenerated plants (T_0_ generation) at 32 weeks post transformation. (**a**) The *MtLYK10* sequences (region of target sites) obtained from the Sanger sequencing of wild-type (WT) plants and from 10 regenerated plants (P1 to P10) were aligned. The two target sites next to the PAM sequences are shown in the wild-type sequence. Mutations at both target sites in P3 resulted in a large deletion. Abbreviations: +, insertion; −, deletion; m, mutation. (**b**) Mutations were identified by cloning the *MtLYK10* amplicons into pMD19-T and Sanger-sequencing plasmid DNA from individual *E. coli* colonies. The arrows in the chromatograms mark the mutation sites.

The variations in *MtLYK10* sequences (single peaks obtained from the plasmid DNA of a single *E. coli* colony) are shown in Figure 4. These sequences were obtained from 10 regenerated plants derived from the transformation of 10 different leaf explants with pISV-CRISPR/*Cas9*P_GmUbi_-sgRNA(*LYK10*). This vector contains sgRNA expression cassettes to edit *MtLYK10* at two different target sites. Mutations were observed at target site 1 for two plants and at target site 2 for four plants. In three plants (P1, P8, and P9), the open reading frame of *MtLYK10* (exon 1) was disrupted. In one plant (P3), mutations at both target sites caused a deletion of 654 bp. Accordingly, the analysis of amplified *MtLYK10* DNA from P3 by agarose gel electrophoresis revealed two bands (Supplementary Materials, Figure S3). Overall, six mutations in the *MtLYK10* gene were obtained at the two target sites, resulting in an editing efficiency of 2/10 (=20%) for target site 1 and 4/10 (=40%) for target site 2.

**Figure 5:**
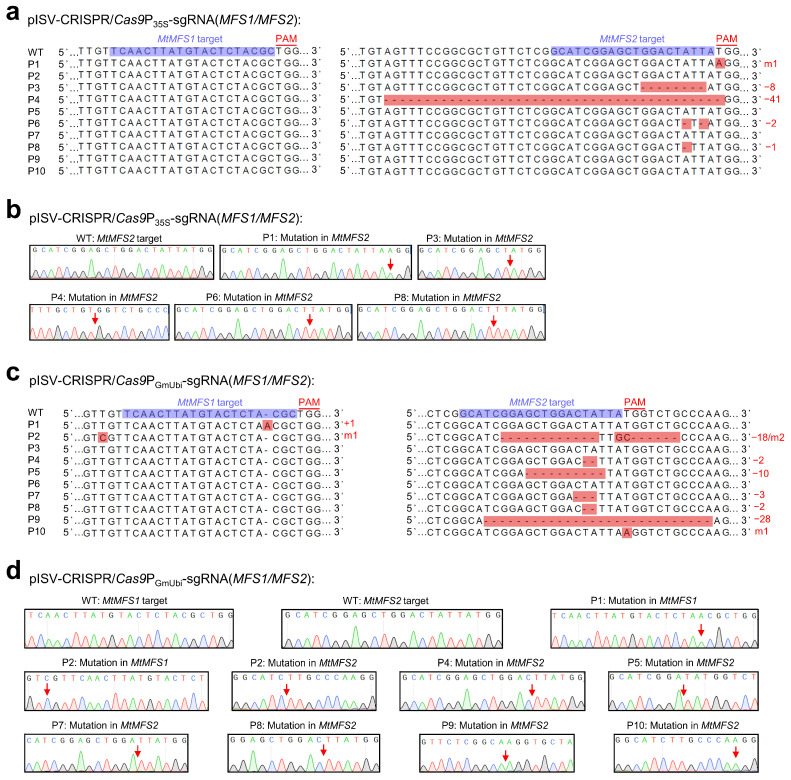
CRISPR/Cas9-mediated editing of *MtMFS1* and *MtMFS2*. *M. truncatula* leaf explants were transformed with *A. tumefaciens* carrying either pISV-CRISPR/*Cas9*P_35S_-sgRNA(*MFS1*/*MFS2*) or pISV-CRISPR/*Cas9*P_GmUbi_-sgRNA(*MFS1*/*MFS2*). Leaf DNA was extracted from wild-type plants and regenerated plants (T_0_ generation) at 32 weeks post transformation. (**a**) *MtMFS1* and *MtMFS2* sequences (region of target sites) obtained from wild-type (WT) plants and from 10 regenerated plants (P1 to P10) transformed with pISV-CRISPR/*Cas9*P_35S_-sgRNA(*MFS1*/*MFS2*). The sequences obtained from Sanger sequencing results (double peaks in mutant plants) were aligned. The target sites next to the PAM sequence are shown in the wild-type sequences. (**b**) Confirmation of identified mutations by cloning the *MtMFS1* and *MtMFS2* amplicons into pMD19-T and Sanger-sequencing plasmid DNA from individual *E. coli* colonies. The arrows in the chromatograms mark the mutation sites. (**c**,**d**) Similar sequence analysis was performed for plants transformed with pISV-CRISPR/*Cas9*P_GmUbi_-sgRNA(*MFS1*/*MFS2*). Abbreviations: +, insertion; −, deletion; m, mutation.

The variations in single-peak sequences of the two examined MFS transporter genes in 10 regenerated plants transformed with pISV-CRISPR/*Cas9*P_35S_-sgRNA(*MFS1*/*MFS2*) and pISV-CRISPR/*Cas9*P_GmUbi_-sgRNA(*MFS1*/*MFS2*) are shown in Figure 5. The two vectors contain identical sgRNA sequences (one target site in the *MtMFS1* gene and another target site in the *MtMFS2* gene), but differ with respect to the promoter controlling *Cas9p* expression (CaMV 35S promoter and GmUbi promoter, respectively). For the plants transformed with pISV-CRISPR/*Cas9*P_35S_-sgRNA(*MFS1*/*MFS2*), no mutation was observed at the target site in *MtMFS1* while mutations at the target site of *MtMFS2* were identified in five plants (50% editing efficiency). Four of these mutations (plants P3, P4, P6, and P8) resulted in a disrupted frameshift of *MtMFS2* (exon 1) (Figure 5a,b). For the plants transformed with pISV-CRISPR/*Cas9*P_GmUbi_-sgRNA(*MFS1*/*MFS2*), two plants showed mutations at the target site of *MtMFS1* (20% editing efficiency) and seven plants at the target site of *MtMFS2* (70% editing efficiency). In one plant (P1), the open reading frame of *MtMFS1* (exon 1) was altered. Four plants (P4, P5, P8, and P9) exhibited sequences with a disrupted open reading frame of *MtMFS2* (exon 1). Furthermore, one plant (P2) showed simultaneous mutations in *MtMFS1* and *MtMFS2*. In this plant, the insertion of a nucleotide disrupted the open reading frame of *MtMFS1* while the mutated *MtMFS2* sequence exhibited a deletion of 18 bp that caused the elimination of six amino acids while the reading frame remained unaffected (Figure 5c,d).

### 3.4. Analysis of Homozygous lyk10 Mutants

**Figure 6:**
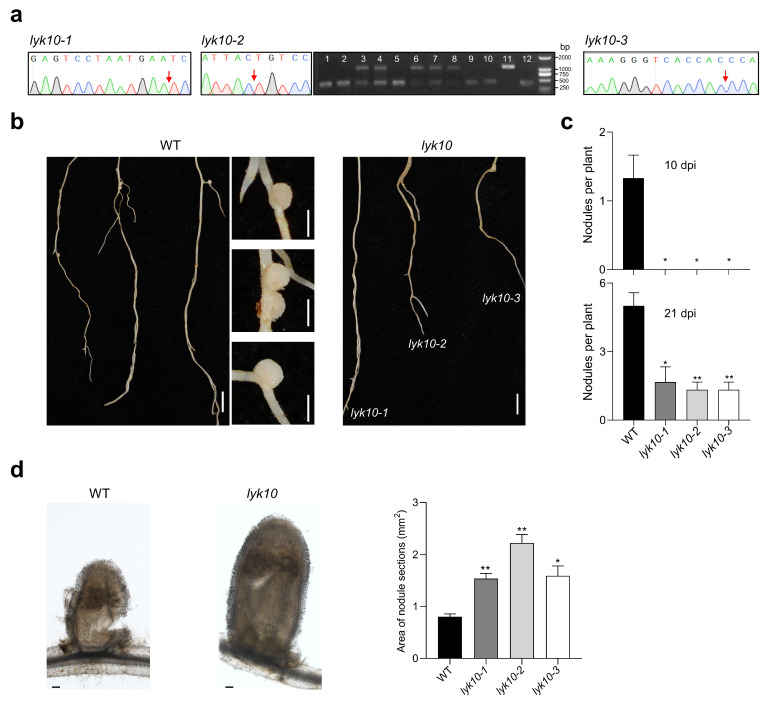
Analysis of nodule formation on the roots of three independent *lyk10* mutant lines. Mutant plants (T_1_ generation of mutant lines *lyk10-1*, *lyk10-2*, and *lyk10-3*) and *M. truncatula* R108 wild-type (WT) plants were inoculated with *S. meliloti* 1021. (**a**) Homozygous mutant plants were identified through the sequencing of *MtLYK10* amplicons (no double peaks at target sites; arrows mark the mutation sites). The amplified DNA of the *lyk10-2* line (derived from P3; see Figure 4) contains a large deletion that could also be visualized using agarose gel electrophoresis. Heterozygous mutant plants showed two bands, whereas for homozygous plants, only a single lower DNA band was detected (lanes 1, 2, 5, 9, 10, and 12). (**b**) Photographs of wild-type and *lyk10* mutant plants harvested at 10 dpi (bar = 1 cm). At this time point, nodules (right images) were formed only on wild-type roots (bar = 1 mm). (**c**) Quantitative analysis of nodule formation at 10 dpi and 21 dpi. (**d**) Left: Example of a longitudinal section of a wild-type nodule and a nodule of the *lyk10-1* mutant (bar = 100 μm). Right: Nodule size as determined by photographing cross-sections of nodules and quantifying the areas with Image J2 software. Data in (**c**,**d**) are presented as mean ± SE (*n* = 3). The asterisks indicate that *lyk10* mutant lines and wild-type plants are significantly different (Student’s *t*-test, * *p* < 0.05, ** *p* < 0.01).

For T_0_ generation plants, the sequencing analysis of *MtLYK10* indicated the presence of frameshift mutations in three independent plants (P1, P3, and P8 in Figure 4). After selfing of these plants, seeds were collected and used for further analysis of the T_1_ generation. *MtLYK10* sequencing data were obtained from leaf DNA of 2-week-old plants. In about one quarter of T_1_ generation plants, no wild-type allele sequences could be observed, indicating the generation of homozygous *MtLYK10* mutant plants (Figure 6a). The mutations in these plants were identical to those of the T_0_ generation plants P1, P3, and P8 (Figure 4). The mutant lines were named *lyk10-1* (derived from P1), *lyk10-2* (derived from P3), and *lyk10-3* (derived from P8). No off-target mutations were identified in these three *lyk10* mutant lines when the DNA of nine genes with predicted off-target sites was sequenced (Supplementary Materials, Table S4). Finally, the identified homozygous *lyk10* mutant and wild-type plants were compared with respect to the formation of root nodules induced by *S. meliloti*. In contrast to wild-type plants, no nodules were observed on the roots of the three mutant lines at 10 dpi (Figure 6b). At 21 dpi, nodules on the roots of mutant plants were observed but their numbers were statistically lower than in wild-type plants (Figure 6c). Surprisingly, the size of the rare nodules in mutant plants was higher than in wild-type plants (Figure 6d). In conclusion, nodule formation was considerably delayed in the three constructed *lyk10* mutant lines.

## 4. Discussion

In this study, we used CRISPR/Cas9 technology to optimize gene editing in the model legume *M. truncatula*. The procedure is based on the transformation of leaf explants with the help of *A. tumefaciens* EHA105 carrying a constructed derivative of pISV-CRISPR/*Cas9*P_GmUbi_. The binary vectors contained a plant codon-optimized *Cas9* gene (*Cas9p*), *Bar*, *DsRed1*, and sgRNA expression cassettes with snRNA promoter sequences of *M. truncatula* (P_MtU6-1_ and P_MtU6-26_). We found that the high transformation and regeneration efficiency of leaf explants under glufosinate selection is probably critical for the construction of mutant plants. Small technical details such as the preparation of surface-sterilized leaf explants (≈1 cm^2^) from healthy leaves and the use of timetin to suppress the growth of agrobacteria were found to be crucial for the regeneration to whole plants. Furthermore, we observed that kinetin (at a concentration of 1 mg·L^−1^ [16]) and the used light conditions [41] are important factors for efficient somatic pro-embryogenesis.

The efficiency of CRISPR/Cas9 systems also depends on strong promoters. Ubiquitin promoters are constitutive promoters that are frequently active in a number of different plants. For example, a ubiquitin promoter from *Lotus japonicus* was routinely used to overexpress genes in *M. truncatula* in previous studies [71,72,73]. When comparing different binary vectors in transformation experiments with *N. benthamiana*, we found the highest mutation frequency at target sites of the co-transformed *GUSPlus* gene for pISV-CRISPR/*Cas9*P_GmUbi_-sgRNA(*GUS*), which contains *Cas9p* under the control of a soybean ubiquitin gene promoter [24,27]. Likewise, the editing efficiency in *M. truncatula* was considerably higher for this vector as compared to the same vector with a CaMV 35S promoter controlling *Cas9p* expression (Figure 5). We therefore recommend using pISV-CRISPR/*Cas9*P_GmUbi_ for future gene editing studies with *M. truncatula*.

To ensure high gene editing efficiency in *M. truncatula*, our sgRNA expression cassettes contained P_MtU6-1_ and P_MtU6-26_, which are snRNA promoters of *M. truncatula*. The P_MtU6-1_ promoter has been used in previous works [26,30,31], while P_MtU6-26_ was found to be as active as P_MtU6-1_ in the current study. The combination of these promoters allowed the simultaneous editing of two target sites in *M. truncatula*. With these promoters, an editing efficiency of up to 70% (per target site) was achieved (Figure 5), which is comparable to or higher than the mutation frequencies reported in previous publications on CRISPR/Cas9-mediated gene editing in *M. truncatula* (e.g., references [24,26,29,31]). Future gene editing in *M. truncatula* could be performed with pISV-CRISPR/*Cas9*P_GmUbi_ containing more than two sgRNA expression cassettes (e.g., by assembling them with the help of *Esp3*I in addition to *Bsa*I). For such constructs, additional *M. truncatula* promoters such as P_MtU6-6_ [31,38] and P_MtU6-8_ [36] could be used. Alternatively, multiple sgRNAs, each separated by RNA cleavage sequences, could be expressed from a single promoter and then processed to sgRNAs, e.g., with the endoribonuclease Csy4 [25].

In general, the probability to obtain a gene knockout mutant increases when the binary vector contains multiple sgRNA sequences for several targets in the same gene. This, however, may also increase the risk of mutations in other genes. In our study, off-target mutations were not identified in the obtained *lyk10* mutant plants. To minimize the risk of off-target mutations, we recommend mutating a given *M. truncatula* gene using a maximum of two sgRNA sequences. Off-target effects could be also reduced by other means, including the use of CRISPR/Cas9 systems with a nuclease-deactivated variant of Cas9 (dCas9) [74].

In our transient transformation experiments with *N. benthamiana*, co-transformation with the two *A. tumefaciens* strains carrying pISV-CRISPR/*Cas9*P_GmUbi_-sgRNA(*GUS*) and pCAMBIA1305 (containing *GUSPlus*) resulted in a surprisingly high number of mutations in the *GUSPlus* gene. It is therefore possible to use *N. benthamiana* leaves as a test system to rapidly verify the functionality and gene editing activity of pISV-CRISPR/*Cas9*P_GmUbi_ derivatives containing sgRNA expression cassettes. *M. truncatula* DNA fragments with target sites could be cloned into pCAMBIA1305 and co-transformation experiments could be performed as described for *GUSPlus* in our study. Results from such a pre-test could provide information on the editing activity of a given sgRNA within a few days (post infiltration with agrobacteria), while the regeneration of leaf explants to whole *M. truncatula* plants takes several months. Indeed, our study shows that the sgRNA sequence chosen for editing *MtMFS1* was suboptimal for unknown reasons and that preliminary transformation experiments with *N. benthamiana* plants are particularly recommended when the mutation of multiple genes is envisaged. The pre-testing of binary vectors with sgRNA expression cassettes could also be performed through the CRISPR/Cas9-mediated reconstitution of a fluorescence protein in *N. benthamiana* [75].

The constructed CRISPR/Cas9 binary vectors contained the fluorescence marker gene *DsRed1*. Accordingly, all regenerated plants were found to be fluorescent when analyzed under red fluorescence conditions. To simplify the removal of the *Cas9p* gene in regenerated *M. truncatula* plants, the *DsRed1* marker will be helpful as non-fluorescent plants can be selected conveniently to obtain transgene-free (T-DNA-free) mutant plants through outcrossing. Furthermore, the *DsRed1* marker allows the convenient identification of transgenic hairy roots obtained through *A. rhizogenes*-mediated transformation. However, the efficiency of CRISPR/Cas9-mediated gene editing in hairy roots and their regeneration to whole *M. truncatula* plants appears to be relatively low in various studies published so far [33,35,36,37,76]. According to our experience, the large-scale regeneration of hairy roots to whole *M. truncatula* plants under glufosinate selection conditions is challenging and less efficient than the regeneration procedure with *A. tumefaciens*-transformed leaf explants described in this study (unpublished observations).

The *MtMFS1* and *MtMFS2* genes of *M. truncatula* mutated in this study encode major facilitator superfamily transporters [77], which are predicted to import or secrete *N*-acetylglucosamine. This sugar has been hypothesized to act as a symbiotic signal in the association between AM fungi and monocots (maize and rice), in which *N*-acetylglucosamine transporters (homologous to *MtMFS1* and *MtMFS2*) have been identified [70]. It is worth mentioning in this context that *M. truncatula* mutants carrying a *Tnt1* retrotransposon in a β-*N*-acetylhexosaminidase gene (*MtHEXO2*) exhibit reduced colonization by AM fungi. MtHEXO2 is an extracellular enzyme that releases *N*-acetylglucosamine from fungal chitooligosaccharide signals [69]. Future research will reveal whether MtMFS1 and MtMFS2 are *N*-acetylglucosamine transporters and whether they are required for the establishment of AM symbiosis.

The *MtLYK10* gene encodes a LysM-type receptor kinase showing homology to exopolysaccharide/glycan receptors of *L. japonicus* involved in nodule formation (LjEPR3 receptor; [78,79,80]) and in symbiosis with AM fungi (LjEPR3a receptor [81]). Our results that homozygous *lyk10* mutants exhibit delayed nodule formation in *M. truncatula* are consistent with findings on a *MtLYK10* mutant reported previously [39]. However, since only a single *Tnt1* retrotransposon insertion mutant was analyzed in that earlier study, the conclusions regarding the symbiotic role of *MtLYK10* had to be interpreted with caution. In our study, three independent *lyk10* mutants inoculated with *S. meliloti* showed a similar symbiotic phenotype. Thus, our results clearly show that *MtLYK10* is a symbiotic gene that positively affects nodule development during early symbiotic stages. Interestingly, when compared to wild-type plants, the size of the rare nodules formed by the three *lyk10* mutant plants was increased, suggesting that MtLYK10 negatively affects nodule size in mature nodules. Alternatively, the increased nodule size was the result of a compensation reaction, as the nodules of poorly nodulated root systems may tend to have a larger volume and more infected cells containing nitrogen-fixing bacteroids. Future experiments are required to investigate whether MtLYK10 can recognize rhizobial glycans such as exopolysaccharide (succinoglycan) [82], cyclic β-glucan [83], and mixed-linkage β-glucan [84].

## 5. Conclusions

This article shows that *MtLYK10* plays a role in nodule symbiosis and that the used CRISPR/Cas9 technology is suitable for efficient gene editing in the model legume *M. truncatula*. The CRISPR/Cas9-mediated generation of mutant lines makes it possible to characterize, with relatively little effort, the function of *M. truncatula* genes for which no or only one gene knockout mutant was obtained in earlier mutagenesis programs [85]. The constructed pISV-CRISPR/*Cas9*P_GmUbi_, pUC-tracrRNA-P_MtU6-1_, and pUC-tracrRNA-P_MtU6-26_ vectors were deposited in the Addgene repository [86] and are available to the scientific community. Our data show that the leaf transformation, glufosinate selection, and regeneration procedures from leaf explants to whole *M. truncatula* plants are highly efficient but require several months to obtain seeds. Future optimization steps should focus on technical parameters that could further standardize and accelerate these processes. Finally, the pre-testing of binary vectors with different sgRNA sequences in *N. benthamiana* should be considered in future experiments, especially if the simultaneous editing of multiple genes is planned.

## Data Availability

The snRNA promoter sequences P_MtU6-1_ and P_MtU6-26_ have been submitted to GenBank (accession numbers OR568513 and OR568514, respectively); plasmids can be requested from C.S.; the plasmids pISV-CRISPR/*Cas9*P_GmUbi_ (Addgene ID 209189), pUC-tracrRNA-P_MtU6-1_ (Addgene ID 209190), and pUC-tracrRNA-P_MtU6-26_ (Addgene ID 209191) are also available via Addgene (https://www.addgene.org; accessed on 7 October 2023).

## Supplementary Materials

The following supporting information can be downloaded at: https://www.mdpi.com/article/10.3390/biology13010053/s1, Table S1. Plasmids used in this study; Table S2. Primers used in this study; Table S3. Target-specific oligonucleotides used in this study; Table S4. Sequence analysis of possible off-target sites in *lyk10* mutants; Figure S1. Illustration of the cloning procedure to generate CRISPR/Cas9 binary vectors with two sgRNA expression cassettes; Figure S2. Identification of *M. truncatula* plants with mutations in *MtLYK10*, *MtMFS1*, and *MtMFS2*; Figure S3. PCR analysis of the P3 plant containing a deletion in *MtLYK10*.
