# Supplementary material for: CRISPR/Cas9-Mediated Generation of Mutant Lines in Medicago truncatula Indicates a Symbiotic Role of MtLYK10 during Nodule Formation
Source: Biology (Basel). 2024 Jan 19;13(1):53. doi: 10.3390/biology13010053 (PMC10812973; doi:10.3390/biology13010053)
Supplement: Supplementary file 1 [file biology-13-00053-s001.zip › biology-2824480-supplementary.pdf]

Supplementary Figures

CRISPR/Cas9-Mediated Generation of Mutant Lines in *Medicago truncatula* Indicates a Symbiotic Role of *MtLYK10* during Nodule Formation

Chun-Xiao Zhang, Ru-Jie Li, Laura Baude, Didier Reinhardt, Zhi-Ping Xie and Christian Staehelin

This file includes:

Figure S1. Illustration of the cloning procedure to generate CRISPR/Cas9 binary vectors with two sgRNA expression cassettes.

Figure S2. Identification of *M. truncatula* plants with mutations in *MtLYK10*, *MtMFS1* and *MtMFS2*.

Figure S3. PCR analysis of the P3 plant containing a deletion in *MtLYK10*.

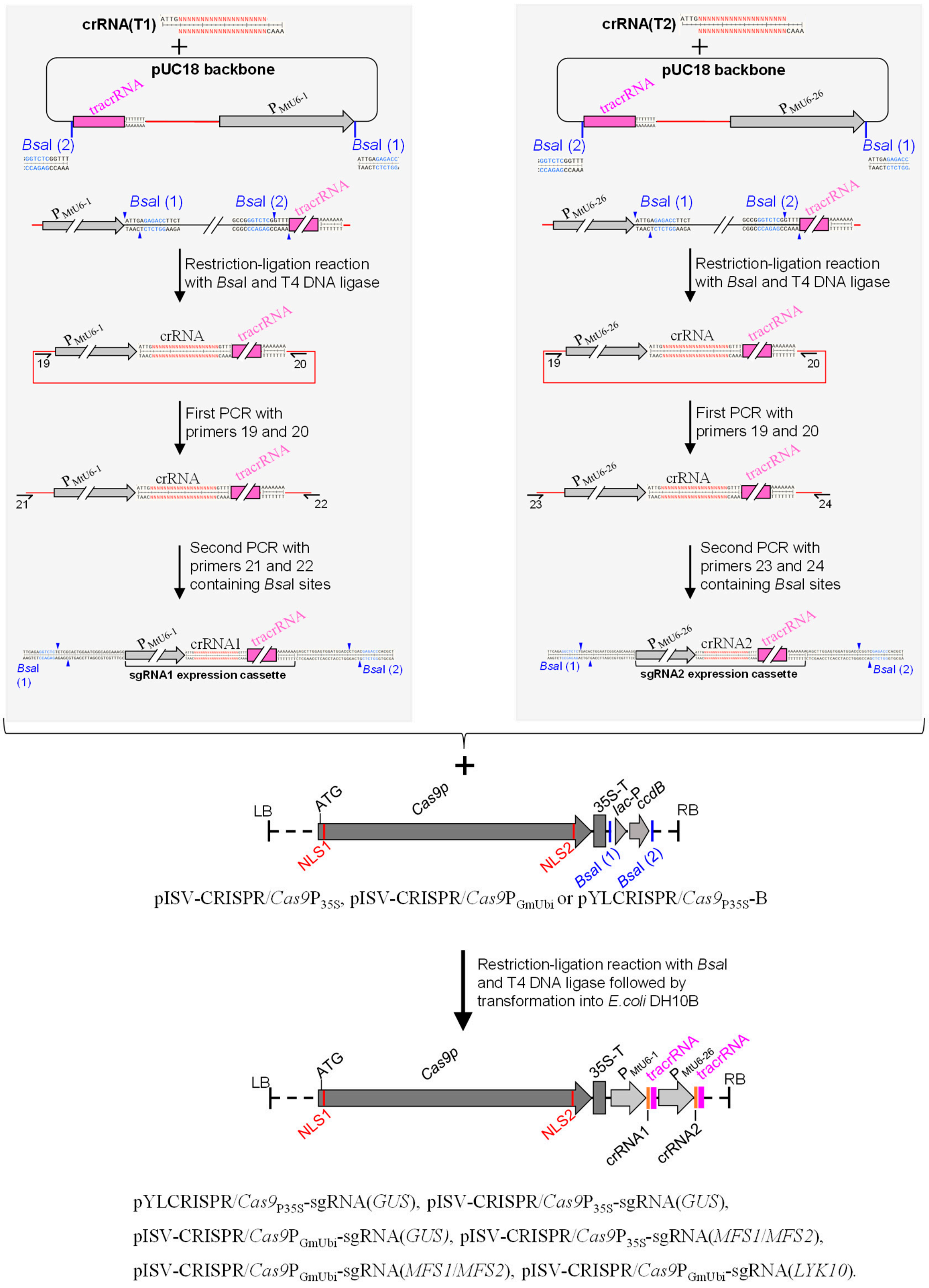

Figure S1. Illustration of the cloning procedure to generate CRISPR/Cas9 binary vectors with two sgRNA expression cassettes. For abbreviations, see legend to Figure 1.

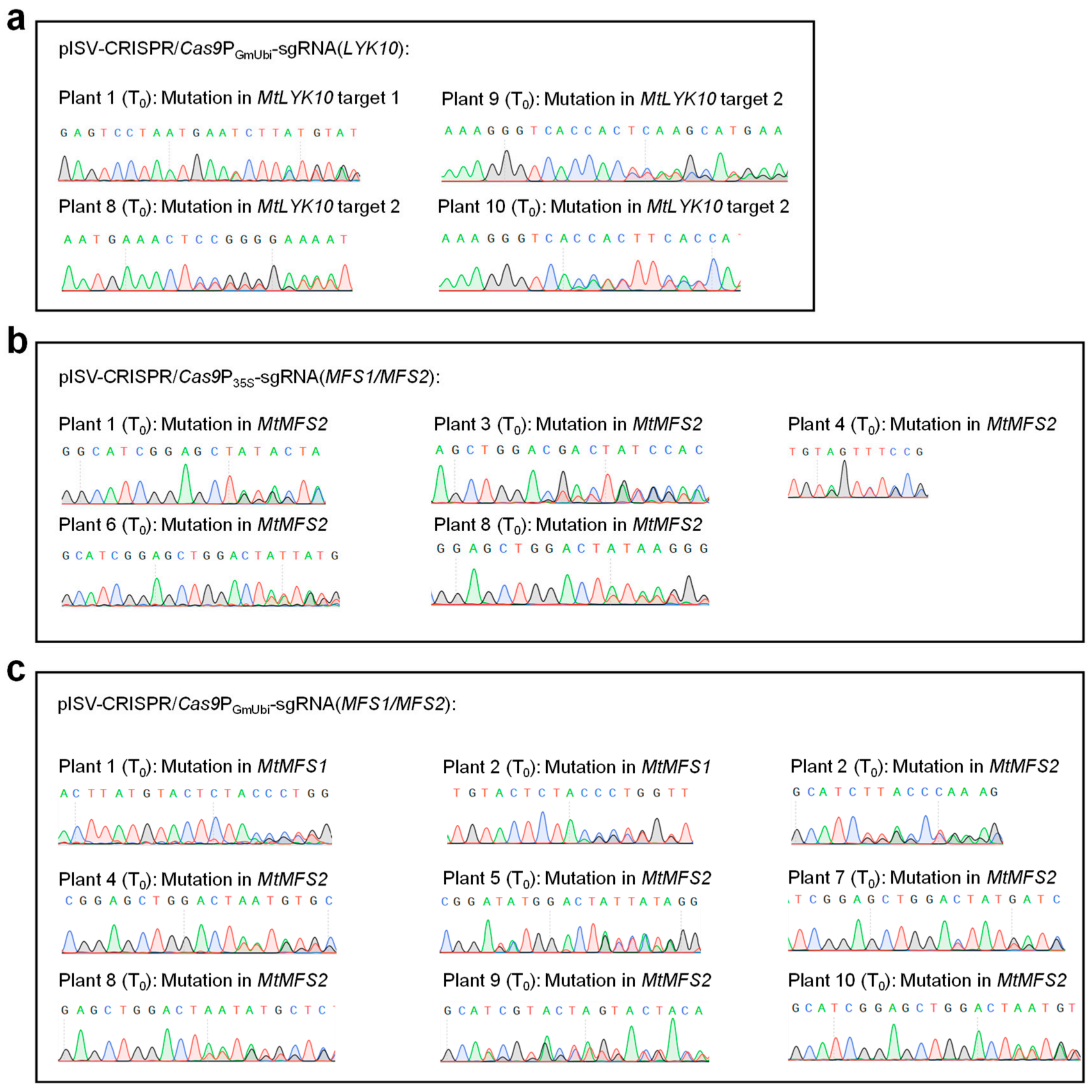

Figure S2. Identification of *M. truncatula* plants with mutations in *MtLYK10*, *MtMFS1* and *MtMFS2*. *M. truncatula* leaf explants were transformed with the indicated binary vectors. Leaf DNA of regenerated plants (T_0 generation) and gene specific primers were used for PCRs and the amplicons were directly sequenced. Sanger sequencing results are shown for plants with mutations at target sites (chromatograms with double peaks). (a) Plants transformed with pISV-CRISPR/*Cas9*P_{GmUbi}-sgRNA(*LYK10*). (b) Plants transformed with pISV-CRISPR/*Cas9*P_{35S}-sgRNA(*MFS1/MFS2*). (c) Plants transformed with pISV-CRISPR/*Cas9*P_{GmUbi}-sgRNA(*MFS1/MFS2*).

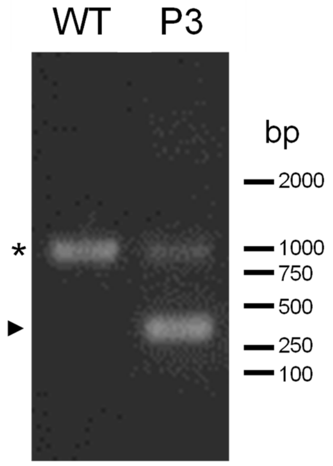

Figure S3. PCR analysis of the P3 plant containing a deletion in *MtLYK10*. *M. truncatula* leaf explants were transformed with pISV-CRISPR/*Cas9*P$_{GmUbi}$-sgRNA (*LYK10*) and a deletion in *MtLYK10* was identified in the P3 plant (T_0 generation) by sequencing. The picture shows an agarose gel with DNA amplified from genomic wild-type and P3 DNA using *MtLYK10* specific primers. Abbreviations: WT, *MtLYK10* DNA amplified from wild-type plants. P3, *MtLYK10* DNA amplified from the heterozygous P3 plant containing a *MtLYK10* wild-type allele (upper band marked by an asterisk) and a mutated *MtLYK10* allele with a 654-bp deletion (lower band marked by an arrowhead).

Supplementary Tables

CRISPR/Cas9-mediated generation of mutant lines in *Medicago truncatula* indicate a symbiotic role of *MtLYK10* during nodule formation

Chun-Xiao Zhang, Ru-Jie Li, Laura Baude, Didier Reinhardt, Zhi-Ping Xie and Christian Staehelin

Table S1. Plasmids used in this study.

Plasmids	Relevant characteristics	Reference/Source
pYLsgRNA-AtU6-1	Vector with a pUC18 backbone, tracrRNA and the AtU6-1 promoter of *A. thaliana*, Amp^r	[43]
pYLCRISPR/*Cas9*P_{35S}-B	Binary vector with a pCAMBIA1300 backbone and *Cas9p/Bar* expression cassettes, Kan^r	[43]
pRT104-*DsRed1*	A 678-bp *DsRed1* fragment PCR-amplified from pX-DR and cloned into pRT104 digested with *Xba*I and *Xho*I, Amp^r	[47]
pISV2678	Derivative of pGPTV-BAR with an enhanced double CaMV 35S promoter. Kan^r	Schultze and Kondorosi; CNRS, Gif-sur-Yvette, France
pUC-tracrRNA-P_{MtU6-1}	Vector with a pUC18 backbone, tracrRNA and the MtU6-1 promoter of *M. truncatula* R108, Amp^r	This study
pUC-tracrRNA-$P_{MtU6-26}$	Vector with a pUC18 backbone, tracrRNA and the MtU6-1 promoter of *M. truncatula* R108, Amp^r	This study

pMD19-P_{Gmubi}	P_{Gmubi} (ubiquitin gene promoter of *G. max* cv. William 82) cloned into pMD-19, Amp^r	This study
pMD19- *DsRed1*	*DsRed1* cassette from pRT104-*DsRed1* cloned into pMD-19, Amp^r	This study
pMD19- *DsRed1*(ΔB)	Point mutated vector of pMD19-*DsRed1* without *Bsa*I (ΔB) restriction site, Amp^r	This study
pISV-*DsRed1*(ΔB)	*DsRed1*(ΔB) expression cassette cloned into the *Hind*III site of pISV2678, Kan^r	This study
pBS-*Cas9-ccdB*	pBluescript II SK (+) derivative carrying *cas9p* and *ccdB* sequences from pYLCRISPR/*Cas9*P_{35S}-B, Amp^r	This study
pISV-CRISPR/*Cas9*P_{35S}	pISV-*DsRed1*(ΔB) derivative carrying *Cas9p* and *ccdB* sequences from pBS-*Cas9-ccdB*, Kan^r	This study
pISV-CRISPR/*Cas9*P_{GmUbi}	pISV-*DsRed1*(ΔB) derivative carrying P_{Gmubi}, the *cas9p* coding sequence and a *ccdB* expression cassette, Kan^r	This study
pYLCRISPR/Cas9$_{P35S}$-sgRNA(*GUS*)	pYLCRISPR/*Cas9*P_{35S}-B derivative carrying two sgRNA expression cassettes for *GUSPlus* editing, Kan^r	This study
pISV-CRISPR/*Cas9*P_{35S}-sgRNA(*GUS*)	pISV-CRISPR/*Cas9*P_{35S} derivative carrying two sgRNA expression cassettes for *GUSPlus* editing, Kan^r	This study
pISV-CRISPR/*Cas9*P_{GmUbi}-sgRNA(*GUS*)	pISV-CRISPR/*Cas9*P_{GmUbi} derivative carrying two sgRNA expression cassettes for *GUSPlus* editing, Kan^r	This study
pISV-CRISPR/*Cas9*P_{35S}-sgRNA(*MFS1/MFS2*)	pISV-CRISPR/*Cas9*P_{35S} derivative carrying a sgRNA expression cassette for *MtMFS1* editing and a sgRNA expression cassette for *MtMFS2* editing, Kan^r	This study

pISV-CRISPR/*Cas9*P_{GmUbi}-sgRNA(*MFS1/MFS2*)	pISV-CRISPR/*Cas9*P_{GmUbi} derivative carrying a sgRNA expression cassette for *MtMFS1* editing and a sgRNA expression cassette for *MtMFS2* editing, Kan^r	This study
pISV-CRISPR/*Cas9*P_{GmUbi}-sgRNA(*LYK10*)	pISV-CRISPR/*Cas9*P_{GmUbi} derivative carrying two sgRNA expression cassettes for *MtLYK10* editing, Kan^r	This study

Table S2. Primers used in this study.

Primers	Sequences (5'→3')	Restriction sites	Description	Amplicon (bp)
1	GATATCGAATTCCTGCAG**CCCGGG**TCTCCACTGACGTAAGGG	*Sma*I	Amplification of *Cas9p* with nuclear localization signals and a terminator sequence from pYLCRISPR/Cas9P35S-B	4668
2	CCGGTCAACATGTGGAGCACGACCGCGTCACTGGATTTTGGTT			
3	AACCAAAATCCAGTGACGCGGTCGTGCTCCACATGTTGACCGG		Amplification of a *ccdB* expression cassette from pYLCRISPR/Cas9P35S-B	765
4	CCGCGGTGGC**GGCCGC**TCTAGATAGCTCGAGAGGCGCGCCA	*Not*I		
5	TCGATTCTAGA**GAATTC**CC**TTAATTAA**GGGCCCAATATAACAACGACGTCG	*EcoRI*, *Pac*I	Amplification of P_{Gmubi} from genomic DNA of *G. max* cv. Willams 82	917
6	TCCCTTACGTCAGTGGAGA**TTAATTAA**GGCTGTCGAGTCAACAATCACAGATAA	*Pac*I		
7	GCTTGCGGCAGCGTGAAGCTTAACATGGTGGAGCACGACACTC		Amplification of a *DsRed1* cassette from pRT104-*DsRed1*	1316
8	GGACCTGCAGGCATGCAAGCTTGATTTTGGTTTTAGGAATTAG			

9	GACGGCGTGCTGAAGGGCGAAACACACAAGGCC CTGAAGC		Amplification of pMD19-*DsRed1(*ΔB*)*	4008
10	GCTTCAGGGCCTTGTGTGTTTCGCCCTTCAGCACG CCGTC			
11	AACATCGATTCTAGA**GAATTC**CC**TTAATTAA**TCT CCACTGACGTAAGGGATG	*EcoR*I, *Pac*I	Amplification of a large DNA fragment containing *Cas9p* and *ccdB*	5466
12	GAGCCCGGGGTCGAC**GAATTC**TAGCTCGAGAGG CGCGCCAATG	*EcoR*I		
13	TCCTTTGCTGCCGATTCCACAGG		Amplification of DNA containing pUC18 and tracrRNA from pYLsgRNA-AtU6-1	2846
14	ATTGAGAGACCTCTGAAGATAAC			
15	GTGGAATCGGCAGCAAAGGAATCCAACATTTCAC TTGAGTTAAC		Amplification of P_{MtU6-1} from genomic DNA of *M. truncatula* R108	541
16	TATCTTCAGAGGTCTCTCAATAAACCCTGCTGTTC GTCTAGGC			
17	GTGGAATCGGCAGCAAAGGATAGGAAATTTTGCT GGGAAAC		Amplification of $P_{MtU6-26}$ from genomic DNA of *M. truncatula* R108	536
18	TATCTTCAGAGGTCTCTCAATAAGCCATTGTGCTC TGTGACG			
19	CTCCGTTTTACCTGTGGAATCG		Amplification of sgRNA expression cassettes	634
20	CGGAGGAAAATTCCATCCAC			
21	TTCAGA**GGTCTC**TCTCGCACTGGAATCGGCAGCA AAGG	*Bsa*I	Amplification of sgRNA expression cassettes with *Bsa*I sites	673
22	AGCGTG**GGTCTC**GTCAGGGTCCATCCACTCCAAG CTC	*Bsa*I		
23	TTCAGA**GGTCTC**TCTGACACTGGAATCGGCAGCA AAGG	*Bsa*I	Amplification of sgRNA expression cassettes with *Bsa*I sites	677

24	AGCGTG**GGTCTC**GACCGGGTCCATCCACTCCAAG CTC	*Bsa*I	
25<br>26	TCGGGTACAGACTAGTTCGT<br>CTTCGAATGGCAGGAATCCG	Confirmation of CRISPR/Cas9 binary vectors containing sgRNA for *GUSPlus*	670
27<br>28	TCAACTTATGTACTCTACGC<br>TAATAGTCCAGCTCCGATGC	Confirmation CRISPR/Cas9 binary vectors containing sgRNA of *MFS1/MFS2*	670
29<br>30	GAGTCCTAATGAAACTTCTG<br>TGGTAAAGTGGTGACCCTTT	Confirmation of pISV-CRISPR/*Cas9P*$_{\mathrm{GmUbi}}$-sgRNA(*LYK10*)	670
31<br>32	CATGGTAGATCTGAGGGTAAATTTC<br>TTCGGAATCTCCACGTTACC	Detection of mutations in *GUSPlus*	940
33<br>34	ATGGGTTCAGCTGAAAATGTAGAATC<br>TACCTGCTAAGAACTTCAGAGTC	Detection of mutations in *MFS1*	1169
35<br>36	ATGGGTGGTGTCGTTGAATATGAAG<br>CTGCTAAGAACTTCAGAGTCATTAGC	Detection of mutations in *MFS2*	1166
37<br>38	ATGGCTTCTCTAATTCAACTTGTTTC<br>GTGACATTGCTTTCAGTAATTACC	Detection of mutations in *LYK10*	1000
39<br>40	ATGAGCCCAGAACGACGCCCG<br>TCAGATCTCGGTGACGGGCAGG	Detection of the *Bar* gene in transformed plants	552
41<br>42	GGAGGCCATAGTGGAATTTTAGGG<br>CATGGTTGTCACTACAATAGAGCCC	Analysis of possible off-target mutations	376
43<br>44	GGATCCTTTTTCGTGGCTAGTGG<br>GCCAGACAGCAACTTTTAGAGTTAGC	Analysis of possible off-target mutations	467
45<br>46	CTCTACACCAAGAGACTGGACTGG<br>AGACATGCTAGTTCCAGATACCATCG	Analysis of possible off-target mutations	391
47	GCAGGAACTCCAAGCTTACACAC	Analysis of possible off-target mutations	420

48	GCTATATTGACTACACGTGTTAGCCG		
49	CCAAATGAGTTACAACAGAGTTAGCAGC	Analysis of possible off-target mutations	359
50	CCAAACAATCTGCAATAGCTTCAGC		
51	ATGGGAACCCTCGGAAGAGC	Analysis of possible off-target mutations	368
52	CATACCCATAAAGCATTACTACGACTACC		
53	GCTTCGAGAATGGCAGGTAACTC	Analysis of possible off-target mutations	471
54	CCTTAAAGGGTCATAATCACTATCGC		
55	GGTTGAAACTAATGCTGGGACATCG	Analysis of possible off-target mutations	395
56	GCAGCACAGTGCACTAAAGCTC		
57	TGGCATACATGTCGATCAATGACG	Analysis of possible off-target mutations	426
58	CGACGATGATTTCAGCGTATGTG		

Table S3. Target-specific oligonucleotides used in this study.

Name	Sequences (5’→3’)	Description	Length (bp)
T-F(*GUS*)-1	attgTCGGGTACAGACTAGTTCGT	*GUSPlus* target 1	20
T-R(*GUS*)-1	aaacACGAACTAGTCTGTACCCGA		
T-F(*GUS*)-2	attgCGGATTCCTGCCATTCGAAG	*GUSPlus* target 2	20
T-R(*GUS*)-2	aaacCTTCGAATGGCAGGAATCCG		
T-F(*MtMFS1*)-1	attgTCAACTTATGTACTCTACGC	*MtMFS1* target 1	20
T-R(*MtMFS1*)-1	aaacGCGTAGAGTACATAAGTTGA		
T-F(*MtMFS2*)-2	attgGCATCGGAGCTGGACTATTA	*MtMFS2* target 2	20
T-R(*MtMFS2*)-2	aaacTAATAGTCCAGCTCCGATGC		
T-F(*MtLYK10*)-1	attgGAGTCCTAATGAAACTTCTG	*MtLYK10* target 1	20
T-R(*MtLYK10*)-1	aaacCAGAAGTTTCATTAGGACTC		

T-F(*MtLYK10*)-2	attgAAAGGGTCACCACTTTACCA	*MtLYK10* target 2	20
T-R(*MtLYK10*)-2	aaacTGGTAAAGTGGTGACCCTTT		

Table S4. Sequence analysis of possible off-target sites in *lyk10* mutants.

Analyzed mutant	Predicted off-target gene	Predicted off-target site	Used primers	Amplicon length (bp)	Mutations in obtained sequences
lyk10-1	MTR_2g063570	AAGTCCTAATGGAAATTCTG AGG *	41, 42	376	No
lyk10-2	MTR_2g063570				No
lyk10-3	MTR_2g063570				No
lyk10-1	MTR_7g029350	ACGTGCTAATGAAACTTCAG TGG *	43, 44	467	No
lyk10-2	MTR_7g029350				No
lyk10-3	MTR_7g029350				No
lyk10-1	MTR_4g127480	GAGTCCAAATGGAACTGATG AGG *	45, 46	391	No
lyk10-2	MTR_4g127480				No
lyk10-3	MTR_4g127480				No
lyk10-1	MTR_2g069950	CAGTCCTAATGAAAATGATT TGG *	47, 48	420	No
lyk10-2	MTR_2g069950				No
lyk10-3	MTR_2g069950				No
lyk10-1	MTR_5g024910	AGAGGATCATCATTTTACCA TGG **	49, 50	359	No
lyk10-2	MTR_5g024910				No
lyk10-3	MTR_5g024910				No
lyk10-1	MTR_3g112020	AAAGGGTCATCAATTTGGTA TGG **	51, 52	368	No
lyk10-2	MTR_3g112020				No
lyk10-3	MTR_3g112020				No
lyk10-1	MTR_1g054795	AAAGTGTCACCTCTTTGCGA TAG **	53, 54	471	No

lyk10-2	MTR_1g054795				No
lyk10-3	MTR_1g054795				No
lyk10-1	MTR_4g116370	AAAAGGTCACCATTTTACCT TCC **	55, 56	395	No
lyk10-2	MTR_4g116370				No
lyk10-3	MTR_4g116370				No
lyk10-1	MTR_7g056073	AAAGCTTCACCACTTCACCA TAT **	57, 58	426	No
lyk10-2	MTR_7g056073				No
lyk10-3	MTR_7g056073				No

* Results from http://skl.scau.edu.cn/offtarget/ using GAGTCCTAATGAAACTTCTGTGG (target 1) in *MtLYK10* as query sequence.

** Results from http://skl.scau.edu.cn/offtarget/ using AAAGGGTCACCACTTTACCATGG (target 2) in *MtLYK10* as query sequence.
